# Synthesis and applications of [60]fullerene nanoconjugate with 5-aminolevulinic acid and its glycoconjugate as drug delivery vehicles†

**DOI:** 10.1039/d1ra08499b

**Published:** 2022-02-22

**Authors:** Maciej Serda, Robert Gawecki, Mateusz Dulski, Mieczysław Sajewicz, Ewa Talik, Magdalena Szubka, Maciej Zubko, Katarzyna Malarz, Anna Mrozek-Wilczkiewicz, Robert Musioł

**Affiliations:** Institute of Chemistry, University of Silesia in Katowice Szkolna 9 40-006 Katowice Poland maciej.serda@us.edu.pl +48322599978 +48323591545; Silesian Center for Education and Interdisciplinary Research 75 Pulku Piechoty 1a 41-500 Chorzow Poland; A. Chełkowski Institute of Physics, University of Silesia 75 Pulku Piechoty 1 41-500 Chorzow Poland; Institute of Materials Science, University of Silesia in Katowice 75 Pulku Piechoty, 1A Chorzow 41-500 Poland; Department of Physics, Faculty of Science, University of Hradec Králové Rokitanského 62 500 03 Hradec Králové Czech Republic

## Abstract

The 5-aminolevulinic acid (5-ALA) prodrug is widely used in clinical applications, primarily for skin cancer treatments and to visualize brain tumors in neurosurgery. Unfortunately, its applications are limited by unfavorable pharmacological properties, especially low lipophilicity; therefore, efficient nanovehicles are needed. For this purpose, we synthesized and characterized two novel water-soluble fullerene nanomaterials containing 5-ALA and d-glucuronic acid components. Their physicochemical properties were investigated using NMR, XPS, ESI mass spectrometry, as well as TEM and SEM techniques. In addition, HPLC and fluorescence measurements were performed to evaluate the biological activity of the fullerene nanomaterials in 5-ALA delivery and photodynamic therapy (PDT); additional detection of selected mRNA targets was carried out using the qRT-PCR methodology. The cellular response to the [60]fullerene conjugates resulted in increased levels of *ABCG2* and *PEPT-1* genes, as determined by qRT-PCR analysis. Therefore, we designed a combination PDT approach based on two fullerene materials, C_60_-ALA and C_60_-ALA-GA, along with the ABCG2 inhibitor Ko143.

## Abstract 2

The 5-aminolevulinic acid (5-ALA) prodrug is widely used in clinical applications, primarily for skin cancer treatments and to visualize brain tumors in neurosurgery.

## Graphical abstract

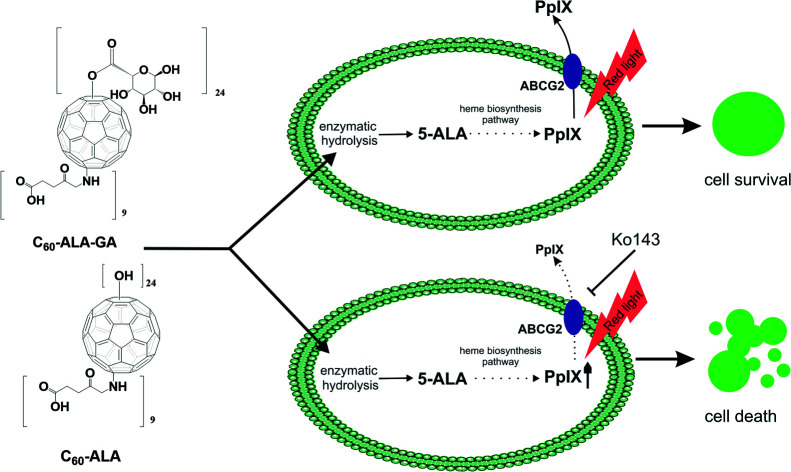

## Introduction

**Fig. 1:**
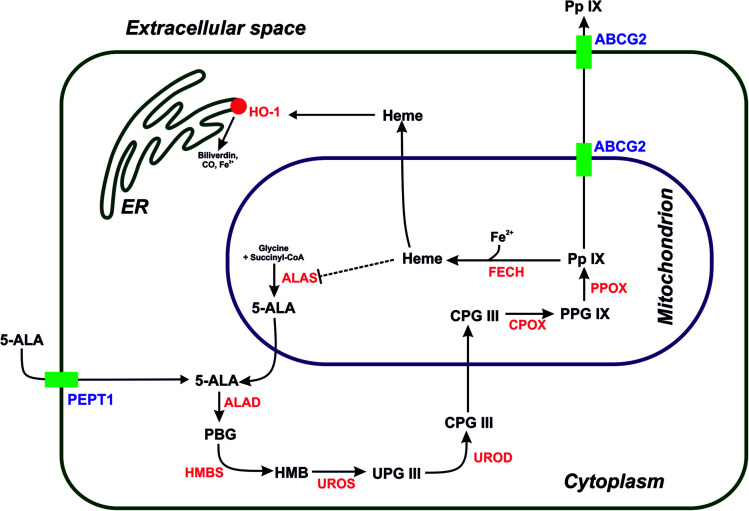
Heme biosynthesis and degradation pathway including selected transporters involved in the flux of intermediates within the pathway. 5-ALA – 5-aminolevulinic acid, PBG – porphobilinogen, HMB – hydroxymethylbilane, UPG III – uroporphyrinogen III, CPG III – coproporphyrinogen III, PPG IX – protoporphyrinogen IX, PpIX – protoporphyrin IX. Individual enzymes involved in the synthesis and degradation are shown in red: ALAD – aminolaevulinic acid dehydrogenase, HMBS – hydroxymethylbilane synthase, UROS – uroporphyrinogen III synthase, UROD – uroporphyrinogen III decarboxylase, CPOX – coproporphyrinogen-III oxidase, PPOX – protoporphyrinogen oxidase, FECH – ferrochelatase, HO-1 – heme oxygenase-1. The transporters involved in the flux are indicated in purple: PEPT1 – peptide transporter 1, ABCG2 – ATP-binding cassette transporter ABCG2.

Owing to its crucial importance as substrate in the heme biosynthetic pathway, 5-aminolevulinic acid (5-ALA) is widely used in dermatology and oncological applications;^1–3^ this is because, despite not being photoactive, it is a prodrug that generates the cellular photosensitizer protoporphyrin IX (PpIX) *via* a cascade of enzymatic reactions.^4^ Photodynamic therapy using PpIX is a well-established procedure in clinical dermatology, especially for treating actinic keratosis, Bowen's disease, and basal cell carcinomas.^5^ Moreover, in malignant glioma therapy, the PpIX generated after the administration of 5-ALA is used as fluorescence marker, because of its high tumor selectivity.^6^ The fluorescence of PpIX can be used for real-time intraoperative imaging to limit the risk of brain shift during surgery, and was clinically approved for fluorescence-guided surgery (FGS). The main disadvantage of therapies based on 5-ALA is its very high hydrophilicity, which limits its bioavailability to only 50–60% after oral administration.^7^ Several approaches have been used to improve the pharmacokinetics of 5-ALA, with the purpose to activate the biosynthesis of PpIX and achieve better photosensitization. These approaches include the formation of methyl and hexyl esters as well as the conjugation of 5-ALA with polymers and nanomaterials.^8,9^ For example, Yao's group created PEGylated gold nanorods coated with 5-ALA for the multimodal therapy of breast cancer; in addition, multiwalled carbon nanotubes (MWCNT) modified with 5-ALA-loaded poly(amidoamine) (PAMAM) showed good biocompatibility, with a significant increase in the accumulation of 5-ALA in MGC-803 tumor cells.^10,11^ From a medicinal chemistry perspective, the crucial enzymes involved in the heme biosynthetic pathway include ferrochelatase (FECH) and heme oxygenase (OH-1), which are responsible for the transformation of PpIX into heme [inactive in photodynamic therapy (PDT)], as well as for its degradation (Fig. 1). Moreover, the inhibition of the ATP-binding cassette transporter (ABCG2), which is responsible for transferring PpIX from the cytoplasm to the extracellular space and activating the PEPT1 protein may improve the treatment. ABCG2 regulates the uptake of 5-ALA into the cytoplasm from an extracellular medium, which may increase the cytosolic concentration of PpIX and therefore improve 5-ALA-based PDT approaches.^12,13^ Recently, our group reported the successful modification of a 5-ALA-based photodynamic therapy by adding thiosemicarbazones scaffolds acting as iron chelators, thereby increasing the accumulation of PpIX.^14,15^

The [60]fullerene scaffold has been used in nanomedicine as drug delivery tool, transfection agent, and theranostic nanomaterial.^16^ Our group previously reported non-receptor tyrosine kinase inhibitors and gemcitabine-delivery vehicles based on [60]fullerene, and showed its preferential biodistribution in an *in vivo* model of breast cancer.^17–19^ The amino acid derivatives of [60]fullerene have been described in the scientific literature since the early 1990s, when the first reports of their antiviral activity inhibiting HIV protease were published by Wudl's group.^20^ From a synthetic point of view, these derivatives can be obtained using three different approaches, such as the Bingel–Hirsch reaction, the 1,3-dipolar cycloaddition (Prato reaction), and the treatment of a buckyball with the desired amino acid in the presence of sodium hydroxide.^21,22^

Fullerene-based amino acids have been used in several biomedical applications as cationic antibacterial nanomaterials or intracellular delivery vehicles of peptides.^22,23^ The use of saccharides in nanomedicine has been investigated since their first reports, mainly focusing on chitosan and hyaluronic acid.^24^ Glycofullerenes have been first investigated in the pioneering studies of Nierengarten, who revealed their antiviral activity against the Ebola virus.^25^ Our previous work on glycofullerenes indicated that sugar derivatives based on d-glucosamine can cross cell membranes and localize in the nuclear envelope and cytosol.^26^ Previous studies described the use of uronic acid in nanomaterial applications; however, its use was limited to the preparation of coatings of metal nanoparticles acting as MRI/CT contrast agents.^27,28^

**Fig. 2:**
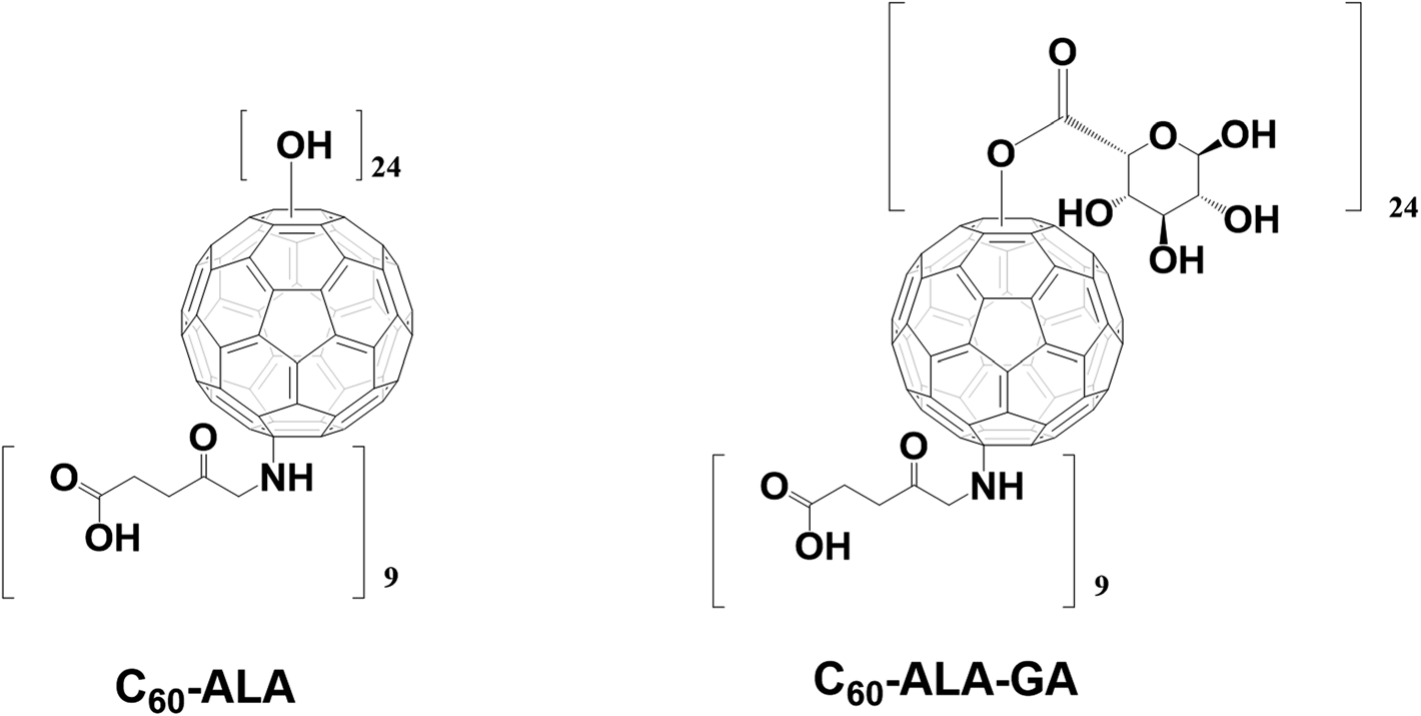
Chemical structures of synthesized fullerene nanomaterials: C_60_-ALA and C_60_-ALA-GA.

The structure of the fullerene nanomaterial C_60_-ALA contains free –OH groups, which are highly beneficial for its further conjugation with sugar-based acids using the 4-dimethylaminopyridine (DMAP)/*N*-ethyl-*N*′-(3-dimethylaminopropyl)carbodiimide (EDCI) system (Fig. 2). This work describes the preparation of two water-soluble fullerene nanomaterials: C_60_-ALA and its glycoconjugate with glucuronic acid (C_60_-ALA-GA). We investigated their physicochemical properties by FT-IR, SEM, TEM, and XPS techniques, and tested their ability to deliver 5-aminolevulinic acid to the cytosolic compartments of three different cancer cell lines (HCT 116, MCF-7, and A549). The cellular levels of PpIX were analyzed by HPLC, in combination with the spectroscopic detection of the cellular fluorescence signal of PpIX. The ability of the synthesized fullerene nanomaterials to influence the four genes of interest (*PEPT1*, *ABCG2*, *HO-1*, and *FECH*) was also tested using the qRT-PCR methodology. In addition, we assessed the photodynamic activity of the synthesized fullerene nanomaterials on five cancer cell lines, and showed how the PDT procedure could be improved by adding the ABCG2 inhibitor Ko143.

## Experimental

### Materials

All compounds used in the experiments were of reagent grade or better, and the solvents were used as received, unless otherwise specified. The following reagents were used as received: 5-ALA hydrochloride (Sigma Aldrich, USA), C_60_ (99.5%, SES RESEARCH, USA), EDCI hydrochloride (Acros Organics, Belgium), ethanol (Avantor, Poland), *N*-hydroxysuccinimide (NHS, Sigma Aldrich, USA), glucuronic acid sodium salt (Acros Organics, Belgium), sodium hydroxide (Avantor, Poland), and Ko143 (Tocris, UK). All solvents were prepared according to the corresponding literature procedures, *i.e.*, treated with a dehydrating agent, distilled, and then used immediately. Nuclear magnetic resonance (NMR) measurements were performed on a Bruker Avance III 500 MHz spectrometer. MS measurements on the [60]fullerene derivative were carried out on an electrospray ionization (ESI) mass spectrometer (Varian 320-MS, USA). The final dialysis purification of the water-soluble fullerene nanomaterials was performed on Microsep™ (Pall Corporation, USA) centrifugal membranes with molecular cutoffs of 1 and 3 kDa. The purities of all compounds were assessed using an Agilent 1260 instrument equipped with a DAAD detector at 260 nm and an RP column (Eclipse Plus C18, 3.5 μm; flow 0.5 mL min^−1^). Fourier transform infrared (FTIR) measurements were carried out using an Agilent Cary 640 spectrometer equipped with a standard source and a DTGS Peltier-cooled detector. The aminofullerene powders were mixed with KBr and their spectra were measured in transmittance mode in the 700–4000 cm^−1^ range. The spectra were recorded with 32 accumulations and a spectral resolution of 4 cm^−1^. The obtained data were analyzed by applying baseline, water, and carbon dioxide corrections. XPS profiles were recorded with a PHI5700/660 Physical Electronics spectrometer with monochromatic Al Kα X-ray radiation (1486.6 eV). The energy of the electrons was measured with a hemispherical analyzer at a resolution of ∼0.3 eV. Photoelectron emissions were measured from a surface area with a diameter of 800 μm, at a take-off angle of 45°. The Multipak (Physical Electronics) program was used for the quantification of the XPS spectra using the peak areas and peak height sensitivity factors. XPS core-level spectra were fitted using the Doniach–Sunjic method.^29^ UV-Vis and fluorescence spectra were measured on V-700 and FP 8500 (JACSO) spectrometers. CHNS elemental analysis was performed with a FlashSmart thermal analyzer (Thermo Fisher Scientific). Fluorescence images were recorded on the CellInsight™ CX7 High Content Analysis Platform (40× magnification) and analyzed with the ImageJ 1.41 software.

### Syntheses

#### Synthesis of C_60_-ALA

A 50 mg (0.07 mmol) amount of fullerene C_60_ was dissolved in 30 mL of freshly distilled toluene using an ultrasonic bath (15 min, purple color). A separate flask of 5-ALA hydrochloride (1.68 g, 10 mmol) was dissolved in 25 mL of deionized water and 5 mL of ethanol (yellow solution). The toluene solution was added to the water solution, and the reaction mixture was heated for 72 h at 82 °C, after which the organic phase became transparent, whereas the color of the water phase changed to brown. The water phase was separated and mixed with Amberlyst-15 (H^+^ form) resin for 1 h until the pH of the fullerene solution was neutral. The water-soluble [60]fullerene derivative was purified using 1 kDa centrifugal membranes; the top layer of the membrane was washed four times with 10 mL of distilled water and passed through a syringe filter (0.2 μm) to sterilize it and remove larger agglomerates. The [60]fullerene nanomaterial was then frozen at −20 °C and freeze-dried, which resulted in a red powder that was stored in a laboratory freezer at −20 °C. The C_60_-ALA product was characterized using NMR and IR spectroscopy, and its structure was confirmed using ESI mass spectrometry.

#### Synthesis of C_60_-ALA-GA

A 243 mg (1 mmol) amount of glucuronic acid sodium salt was dissolved in 10 mL of DI water, keeping the temperature of the solution at approximately 5 °C; then, the conjugation mixture was added (EDCI and NHS, 1 mmol of each component). The reaction mixture was stirred for 30 min at a lower temperature, followed by the addition of a water solution of C_60_-ALA (10 mL, 0.0417 mmol, 90 mg). The reaction mixture was stirred for 24 h at room temperature, and the final glycoconjugate was purified using a 3 kDa centrifugal membrane; the product was then frozen at −20 °C and freeze-dried, giving a red powder that was stored in a laboratory freezer at −20 °C.

### Quantitative HPLC measurements of PpIX

We used a methodology previously reported by the Ogura group, with some modifications.^30^ Briefly, cells were seeded at a density of 500 000 cells and incubated for 24 h. The cells were then incubated with 5-ALA (1 mg mL^−1^) and fullerene derivatives (1 mg mL^−1^) for 24 h in the dark, and untreated cells were used as control. The cells were washed twice with phosphate-buffered saline (PBS) and then treated with 200 μL of 0.1 M NaOH. Next, cell lysates were treated by adding 3 volumes of 1 : 9 (v/v) solution of solvent A (1 M ammonium acetate, 12.5% acetonitrile, v/v) and solvent B (50 mM ammonium acetate, 80% acetonitrile, v/v) to the NaOH-treated cell samples. The prepared samples were centrifuged at 10 000 × *g* for 10 min at 4 °C and analyzed using HPLC. The HPLC measurements were carried out on a Gynkotek system, under the following conditions: T580 pump; GINA50 autosampler; DAAD UVD340U detector; Hilic Kinetex 100 Å column (Phenomenex, Torrance, CA, USA); flow: 0.7 mL min^−1^; MeOH/H_2_O 90 : 1 (v/v); UV wavelength: 400 nm; software: Chromeleon (Thermo Scientific, Waltham, MA, USA). The concentrations of PpIX in the cellular extracts were estimated from calibration curves of the protoporphyrin IX standard (Sigma Aldrich). Calibration curve: *y* = 266.0174*x* − 54.3023 (standard deviation, SD = 16.179, *R*^2^ = 0.9911); limit of detection (LOD): 0.2 μg mL^−1^; limit of quantification (LOQ): 0.66 μg mL^−1^.

### Cell culture methodology

MCF-7, A549, and HCT 116 cell lines were purchased from ATCC. All cell lines were cultured in Dulbecco's Modified Eagle's Medium (DMEM) supplemented with 12% of heat-inactivated fetal bovine serum (FBS) and antibiotics (mixture of penicillin and streptomycin, Gibco). The cells were grown at 37 °C in a humidified atmosphere with 5% CO_2_.

### Cytotoxicity assay

Twenty-four hours before the experiment, the cells were seeded into 96-well plates (Nunc, 5000 cells per well). The next day, the fullerene derivatives (concentration: 1 mg mL^−1^) were added to the plates. After 72 h, the cytotoxicity was determined using the CellTiter 96® AQueous One Solution MTS assay (Promega). For this purpose, after removing the fullerene-containing medium, 100 μL DMEM and 20 μL MTS were added to each well. After 1 h, we measured the absorbance at 490 nm. The absorbance of the untreated control was set as 100%. Each experiment was repeated at least three times.

### Protoporphyrin IX fluorescence intensity assay

The cells were seeded in 96-well plates (Black Clear Bottom, Corning) at a density of 11 000 cells per well for 24 h. Then, we prepared solutions of the fullerene derivatives and 5-ALA (concentration: 1 mg mL^−1^) in a serum- and a phenol red-free medium, and added them to the cells. The incubation time was set to 3 or 24 h in the dark, depending on the experiment. For the experiments with the Ko143 inhibitor, the cells were initially incubated with the inhibitor at a concentration of 5 μM for 1 h; after that, the cells were washed and the fullerene nanomaterials were added. Next, the medium containing the tested compounds was removed and used to wash the cells twice. The fluorescence of PpIX was measured at excitation and emission wavelengths of 407 and 638 nm, respectively. Reduced light conditions protected the PpIX compound from photodegradation. The experiments were repeated at least three times. The PpIX concentration was calculated as a percentage of the 5-ALA one, and the results were expressed as relative fluorescence units (RFU).

### Gene expression assay

All the tested cell lines were seeded in Petri dishes (Nunc, 400 000 cells per 3 cm dish) and incubated for 24 h. After that, the medium was removed, and solutions of 5-ALA (1 mg mL^−1^) and fullerene derivatives (1 mg mL^−1^) were added for 24 h. The next day, the total cellular RNA was isolated using TRIzol reagent (Ambion). cDNA was synthesized with 3 μg of RNA using GoScript™ Reverse Transcriptase kit (Promega) and Oligo(dT)_23_ primers. Quantitative real-time PCR analysis was conducted using a CTX96 Touch™ system (Bio-rad). The reaction mixture contained 1 μL cDNA, SYBR Green Mix (Bio-rad), and the specific primer pair mixture (Sigma Aldrich) designed for each target. The primer pair sequences (Sigma, Table S4, ESI†) were designed using Primer 3. The following procedures were followed: initial denaturation at 95 °C for 20 s; 40 denaturation cycles at 95 °C for 10 s and then extension at 72 °C for 30 s; annealing at primer-specific temperature for 20 s and then at 72 °C for 30 s. The reference housekeeping gene (GAPDH) expression was compared to that of the analyzed target genes using the 2^−ΔΔ*C*_*t*_^ (Livak–Schmittgen) method. The experiments were repeated at least three times.

### Phototoxicity assay

The cells were seeded at a density of 11 000 well for 24 h. Then, they were incubated with 5-ALA and the fullerene derivatives at a concentration of 1 mg mL^−1^ for 3 h in the dark (5% CO_2_ at 37 °C). For the experiments with the Ko143 inhibitor, the cells were incubated with the inhibitor at a concentration of 5 μM for 1 h, after which they were washed and incubated with the tested compounds. Subsequently, the cells were washed three times with serum- and phenol red-free media and irradiated with red light (634 ± 5 nm) at 12 J cm^−2^. The cells were further incubated in the dark under 5% CO_2_ at 37 °C for 24 h. The cell viability was then measured using an MTS assay, as described above (see Cytotoxicity assay paragraph).

### Fluorescence microscopy

The MCF-7 cell line was seeded in glass-bottom Petri dishes (Nunc, 300 000 cells per 3 cm dish) and incubated for 24 h. Then, the cells were incubated with 5-ALA and the fullerene derivatives at a concentration of 1 mg mL^−1^ for 3 h in the dark (5% CO_2_ at 37 °C). For the experiments with the Ko143 inhibitors, the cells were preincubated with the inhibitor at a concentration of 5 μM for 1 h, after which they were washed and treated with the tested compounds. After that, the Hoechst 33342 dye (6.5 μM) was added and the cells were incubated for 15 min. Then, the cells were washed with phenol red-free DMEM; fluorescence images were captured using the CellInsight™ CX7 High Content Analysis Platform (40× magnification) and analyzed with the ImageJ 1.41 software.

### Statistical analysis

The results were expressed as mean ± SD from at least three independent experiments. One-way ANOVA with Tukey *post hoc* test analyses were carried out with the GraphPad Prism v.7.0 software (USA). A *p* value of 0.05 or less was considered statistically significant.

## Results and discussion

**Fig. 3:**
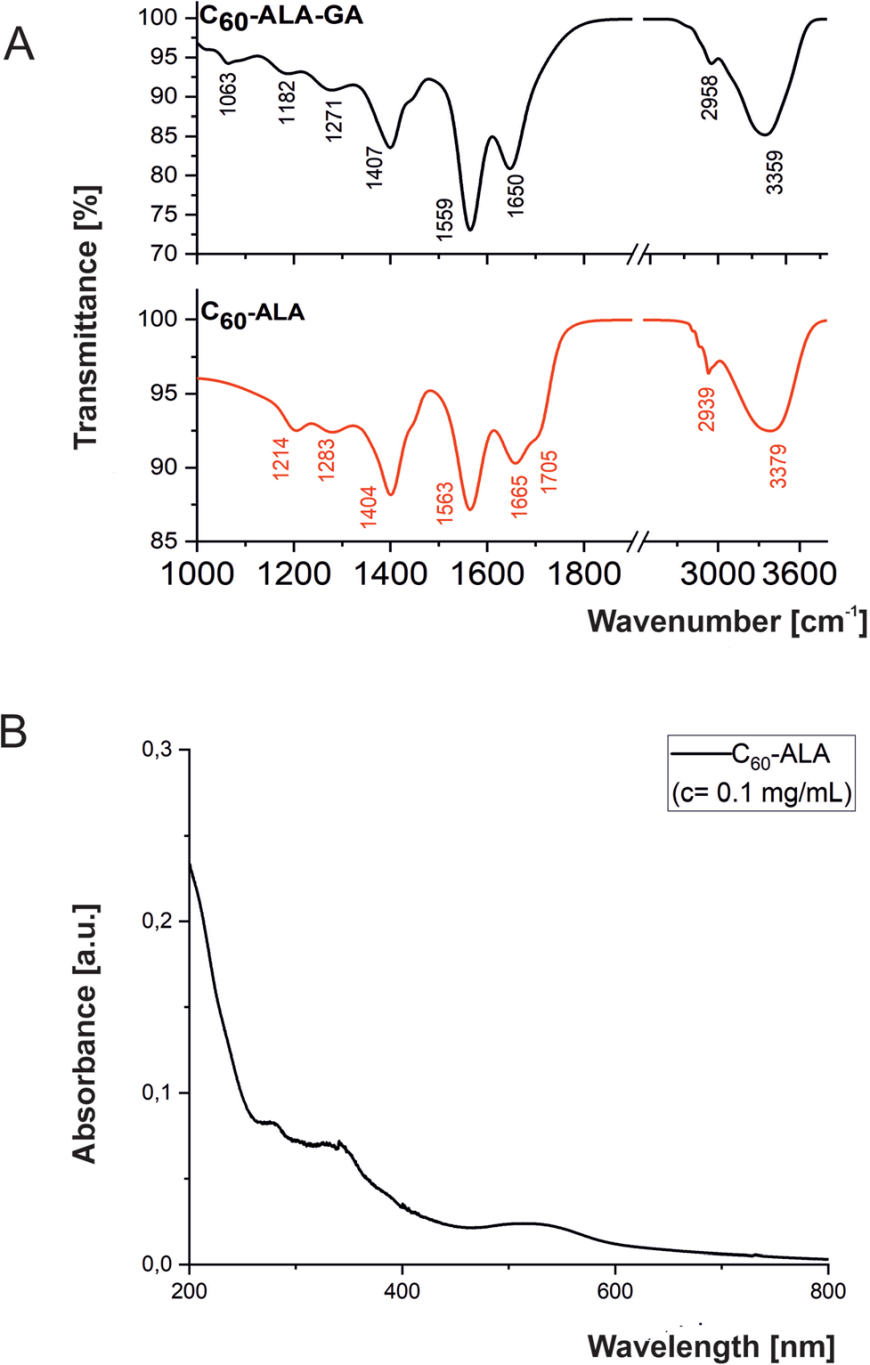
Spectroscopic characterization of C_60_-ALA and C_60_-ALA-GA fullerene nanomaterials. (A) FT-IR spectra of the two nanomaterials. (B) UV-Vis spectrum of C_60_-ALA.

The two water-soluble fullerene nanomaterials were synthesized by adding 5-aminolevulinic acid to the [60]fullerene substrate in the presence of sodium hydroxide in water/ethanol solution (the reaction scheme is shown in Fig. S1, ESI†). In the purification process preceding the membrane dialysis, the C_60_-ALA nanomaterial was treated with an ion-exchange resin (Amberlyst 15, H^+^ form) to ensure the formation of sodium-free OH groups. The ^13^C-NMR spectrum confirmed the successful addition of the 5-aminolevulinic fragments to the fullerene substrate, with characteristic peaks at ∼200 and 180 ppm representing two different carbonyl groups of the 5-ALA molecule. The broadening of the carboxyl group near 180 ppm may be attributed to the dynamics of the terminal –COOH group, which is located away from the buckyball core. The two peaks at 74 and 66 ppm in the ^13^C-NMR spectrum corresponded to the two sp^3^ carbon types (C–O and C–N) present in the C_60_-ALA structure (Fig. S2, ESI†). A previous study reported that the signals of the sp^3^ carbons of the C_60_(OH)_24_ hydroxyfullerenes^31^ and the polyamine derivatives of the buckyball described by Hirsch^32^ fall in that region of the carbon spectrum. ESI mass spectrometry and elemental analysis were used to determine the molecular formula of C_60_-ALA. Based on the carbon to nitrogen (C/N) ratio obtained from the elemental analysis (Table S1, ESI†), we calculated the molecular formula of C_60_-ALA to be C_60_(NHCH_2_COCH_2_CH_2_COOH)_*x*_(OH)_*y*_, where *x* = 8.90, corresponding to nine 5-ALA units attached to the buckyball scaffold. Because the presence of oxygen may also be due to adsorbed water in 5-ALA, we calculated the number of hydroxyl groups (*y* = 24) attached to the C_60_ core from the mass spectrum of C_60_-ALA. The calculated molecular formula of C_60_-ALA was C_105_H_96_N_9_O_51_, corresponding to a molecular mass of 2298 Da. The ESI mass spectrum in Fig. S3† shows a strong signal at 2228 Da, which was assigned to C_60_-ALA molecules with four –OH groups fragmented from the parent structure, likely due to the high voltage applied in the experiments. We performed additional measurements in negative ionization mode (more suitable for carboxylic acids, Fig. S4, ESI†) using a lower voltage setup, and the obtained spectrum still showed the molecular peak at 2297 Da, along with the 2230 Da fragment (20 OH units attached to C_60_). The UV-Vis spectrum of C_60_-ALA, shown in Fig. 3, displays the characteristic profile of a water-soluble fullerene nanomaterial (*c* = 0.1 mg mL^−1^), with three local maxima at ∼280, 340, and 530 nm. The signal at approximately 340 nm was attributed to π-conjugated transitions in the fullerene molecules, whereas that above 500 nm could be assigned to a first-order forbidden transition.^33^ The fluorescence of engineered carbon nanomaterials can provide insight into their cellular uptake; C_60_-ALA showed a weak fluorescence around 470 nm when irradiated at 340 nm (Fig. S10, ESI†). Unfortunately, the observed fluorescence faded at higher wavelengths; therefore, these effects could not be used in the cellular localization experiments.

The infrared spectra of C_60_-ALA and C_60_-ALA-GA presented in Fig. 3 showed similar bands arranged in two regions: (1) 2450–3800 cm^−1^ and (2) 1000–1900 cm^−1^. Interestingly, small differences between the band numbers, intensities, or positions of the two compounds clearly reflected different functional groups anchored to the fullerene surface. Specifically, region 1 was associated with the symmetric and asymmetric stretching modes of CH_*x*_ (*x* = 1, 2) moieties, as well as the stretching vibrations of hydroxyl and amine groups. Furthermore, the position of the hydroxyl band reflected the tendency of the chains anchored to the fullerene surface to form H-bonds of moderate or medium–high strength. In turn, the high value of the full width at half maximum (FWHM) suggested a wide distribution of H-bonds with different donor–acceptor bond lengths. Unfortunately, we could not directly identify the presence of amine groups, because of the strong impact of the hydroxyl units on the hydrogen network. Moreover, a few differences were observed in the shape of the hydroxyl bands of the two studied fullerenes as a result of substitution with sugar derivatives. Differences in the H-bonding character were also observed in region 2, especially at thigh wavenumbers. Here, the two bands centered at 1665 and 1705 cm^−1^ (C_60_-ALA) as well as the single band at 1650 cm^−1^ (C_60_-ALA-GA) corresponded to the stretching vibration of the carbonyl groups (Fig. 3); in particular, the position of the low-lying carbonyl bands resulted from the high impact of the hydrogen bonds, while the second carbonyl band corresponded to less strongly bonded groups. These results suggest that all carbonyl groups were H-bonded in the case of C_60_-ALA-GA, whereas some weakly-bonded C=O groups were also present in C_60_-ALA. This effect was attributed to the sugar substitution, which strongly altered the hydrogen bond network, in good agreement with the above explanation. A similar interpretation of the impact of the H-bonds was obtained from the analysis of the positions of the other bands in the fingerprint regions of C_60_-ALA/C_60_-ALA-GA, *i.e.*, 1563/1559, 1404/1407, 1283/1271, and 1214/1182 cm^−1^. The assignments of the bending modes of secondary amines, the skeletal vibrations of alkane chains, and the deformation modes of CH_*x*_ (*x* = 1, 2) groups were similar in the two fullerenes. In addition, the introduction of sugar derivatives led to the appearance of a new band at 1063 cm^−1^, associated with the CCO stretching vibrations within the sugar ring.

TEM and dynamic light scattering (DLS) experiments were used to analyze in detail the formation of fullerene aggregates in the water solution. As previously reported in our studies and by Wilson's group, water-soluble fullerenes tend to form both smaller and larger aggregates, which remain in a dynamic equilibrium between each other.^17,26^

The studied C_60_-ALA nanomaterial formed relatively larger agglomerates consisting of smaller spherical clusters, as shown in the corresponding bright-field TEM image (Fig. 5). Ten recorded TEM images were analyzed to determine the size of the agglomerates. The distributions shown in Fig. 5 were based on the analysis of the diameters of 297 spherical agglomerates. As shown by the fitted curve colored in red, the shape of the curves closely followed a log-normal distribution. From the fitted curve, the average diameter of the agglomerates was determined to be 19.9(2) nm, with a standard deviation of 5.5(2) nm. These results were compared with the DLS measurements for C_60_-ALA, for which smaller agglomerates (40 ± 6.8 nm) were also observed; however, the corresponding distribution was dominated by larger agglomerates of ∼308 nm (Fig. S5†). Moreover, the glycoconjugated C_60_-ALA-GA fullerene formed only one agglomerate of ∼227 nm size, which might be explained by the additional stability provided by the larger number of –OH groups originating from the d-glucuronic acid fragment (Fig. S7†). To further analyze the stability of the fullerene aggregates, we measured the zeta potentials (Fig. S6 and S8, ESI†). C_60_-ALA-GA had a higher zeta potential (−38.9 mV, Fig. S8†) than the unmodified C_60_-ALA (−26.2 mV, Fig. S6†), supporting the hypothesis that the glycoconjugated fullerene had a higher stability. In order to elucidate the morphology of the synthesized fullerene nanomaterials, we analyzed the structure of C_60_-ALA using SEM. It was previously reported that the highly water-soluble fullerene C_60_-ser formed shell-like arrangements with voids ranging from 100 nm to microns.^17^ Compared with previously reported systems, the present C_60_-ALA fullerene nanomaterial exhibited thicker walls. In addition, we observed the formation of spherical aggregates for this nanomaterial (Fig. 5) and confirmed its hollow structure, with sizes similar to those reported by Lapin *et al.*^17^ (Fig. S11, ESI†). The surface of C_60_-ALA-GA was also examined using SEM (Fig. S12, ESI†). This analysis confirmed the spherical shape of C_60_-ALA-GA aggregates, but their hollow structure could not be clearly observed.

**Fig. 4:**
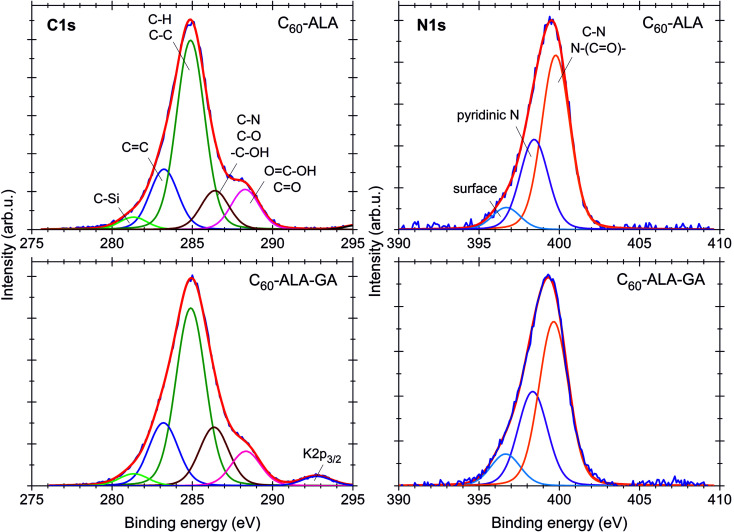
XPS profiles of C_60_-ALA and C_60_-ALA-GA fullerene nanomaterials.

The electronic structure and elemental compositions of the fullerene samples were studied using XPS. Fig. S13† shows the XPS spectra of both fullerene nanomaterials in the wide energy range of 0–1400 eV. Besides the signals related to the main elements forming the compounds, such as C, O, and N, additional signals corresponding to Na, Si, F, K, S, and Cl were observed in the spectrum. These can be considered trace elements originating from synthetic impurities or sample preparation. The atomic concentrations in both [60]fullerene nanomaterials were calculated based on the ratio of each of the components to the sum of all of the elements. Except for H and He, all elements could be detected, with a detection limit of 0.1 at%. The chemical compositions and calculated atomic concentrations of both fullerenes are listed in Table S3 (ESI†). Some differences were found between the atomic concentrations and relative ratios of the single components of the two nanomaterials. The most significant difference was found for the oxygen-containing groups (Table S3, ESI†). The analysis of the C 1s line (Fig. 4) revealed that the numbers of C–O, C–N, and –C–OH bonds (calculated using the peak area ratios relative to the C–C and C–H bonds) increased from 20% (C_60_-ALA) to 35% (C_60_-ALA-GA), while the number of C=O and O–C–OH bonds decreased slightly, from 21% (C_60_-ALA) to 19% (C_60_-ALA-GA). The C 1s, O 1s, and N 1s photoemission lines were used to determine possible chemical bonds between carbon, oxygen, and nitrogen (Fig. 4). The deconvolution of the spectrum revealed that the C 1s peak was composed of five components, corresponding to the carbon atoms in different functional groups. The lowest binding energy signal (281.5 eV) was attributed to the presence of silicon impurities originating from SiC. The second peak at 283.2 eV was related to the C=C bonds in the fullerene core. The most intense component for both samples, at 284.9 eV, was assigned to unoxidized graphite carbon atoms in C–C or C–H groups.^34,35^ The 286.4 eV peak was attributed to oxygen- and nitrogen-containing groups (C–O, C–N, or –C–OH), whereas that at 288.3 eV was assigned to carbonyl and carboxyl (C=O or O=C–OH) groups.^36,37^ The O 1s spectra of both fullerenes (Fig. S14†) revealed the presence of four components, corresponding to the carbonyl (C=O, 531.9 eV), carboxyl (O–C=O, 533.3 eV), and quinone at 530.5 eV. Moreover, the peak at 528.7 eV was attributed to the weakly adsorbed oxygen O_2_/OH^−^. In the N 1s spectra, three components were observed. The peaks located at 399.8 eV and 398.4 eV were ascribed to the C–N or N–(C=O)– bonds and basic nitrogen (pyridinic type), respectively.^38,39^

**Fig. 5:**
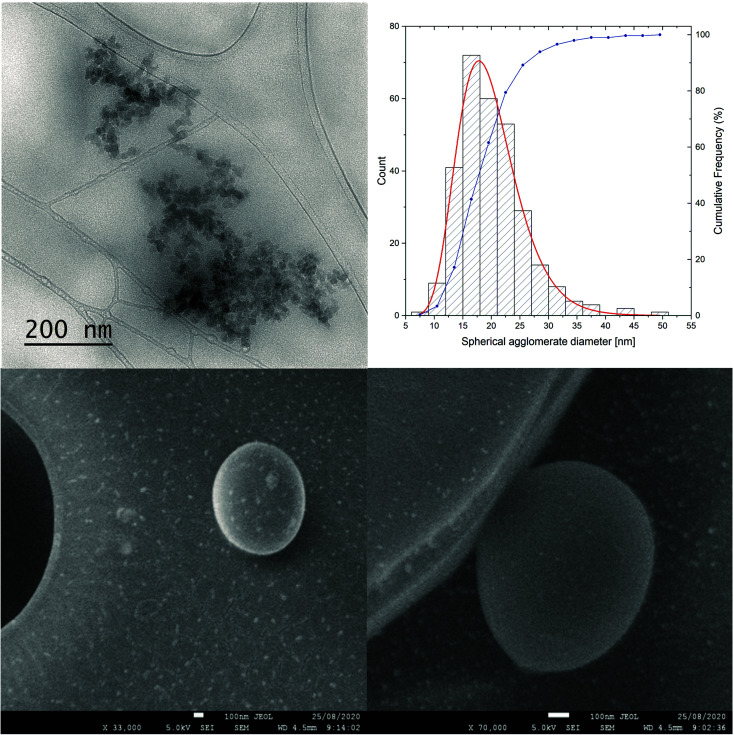
TEM and SEM analyses of C_60_-ALA fullerene nanomaterial.

When developing efficient drug-delivery nanovehicles for zwitterionic compounds such as the 5-aminolevulinic acid considered in this study, several strict criteria must be met to guarantee successful at the *in vitro* level as well as in complex animal models.^40^ The most common way to improve the pharmacological properties of 5-ALA is the formation of esters (ethyl and propyl), which are able to deliver the compound to the cytosol and further release it by enzymatic cleavage. In most cases, esterases and proteases are used to cleave drugs from an engineered nanoconjugate. In this work, we faced a completely different situation because the developed [60]fullerene derivatives contained carbon–nitrogen (C–N) bonds between the buckyball carbon atoms and the amine groups of 5-ALA. Therefore, the question was whether the carbon nanomaterials could be cleaved from essential amino acids *via* cellular enzymatic mechanisms. Fortunately, there are several enzymes present in cells that can catalyze this type of cleavage (deamination). They are classified as carbon-nitrogen lyases (4.3 subcategory of the enzyme classification system), and some of them use amino acids and dipeptides as substrates (*i.e.*, argininosuccinate lyase). However, the direct detection of the enzyme involved in the hydrolysis process of fullerene nanomaterials was not possible and would require additional computational and experimental studies; this is primarily because the interactions between the [60]fullerene scaffold and proteins are complex and associated with the formation of the protein corona adsorbed on the fullerene surface.^41,42^

To address the hydrolysis issues discussed above, we used two independent detection methods for the 5-ALA primary metabolite protoporphyrin IX, namely HPLC (on cellular extracts) and *in vitro* fluorescence detection. This approach is based on the assumption that, if C_60_-ALA and C_60_-ALA-GA were hydrolyzed, their primary metabolite could be easily detected and quantified using these analytical methods. In the chromatographic approach, we incubated the selected cancer cell lines with the water-soluble [60]fullerene derivatives for 24 h; then, they were pretreated using sodium hydroxide, and the final lysis solution (ammonium acetate/acetonitrile) was subsequently centrifuged. HPLC analysis of the generated PpIX was performed at 400 nm by detecting the characteristic Soret bands of the porphyrin core. Representative chromatograms of MCF-7 breast cancer cells, untreated and treated with 5-ALA and C_60_-ALA, are shown in Fig. S9 (ESI†). The quantitative analysis of the HPLC detection of PpIX is shown in Table S2 in the ESI;† the data were normalized to the number of millimoles of substrate used in the experiment. For the analytical calculations, we assumed that the maximum number of cleaved 5-aminolevulinic units was nine, based on the results of the previous elemental analysis. C_60_-ALA-GA emerged as the most effective drug-delivery vehicle, although both fullerene carriers delivered 5-ALA into the cytosol more efficiently, leading to higher detected levels of PpIX.

**Fig. 6:**
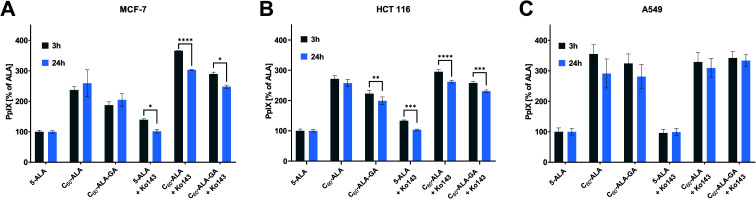
Fluorescence of accumulated PpIX after 3 and 24 h of incubation with tested compounds on MCF-7 (A), HCT 116 (B), and A549 (C) cell lines. Data were analyzed using one-way ANOVA with Tukey *post hoc* test. *p < 0.05; **p < 0.01; ***p < 0.001; ****p < 0.0001.

**Fig. 7:**
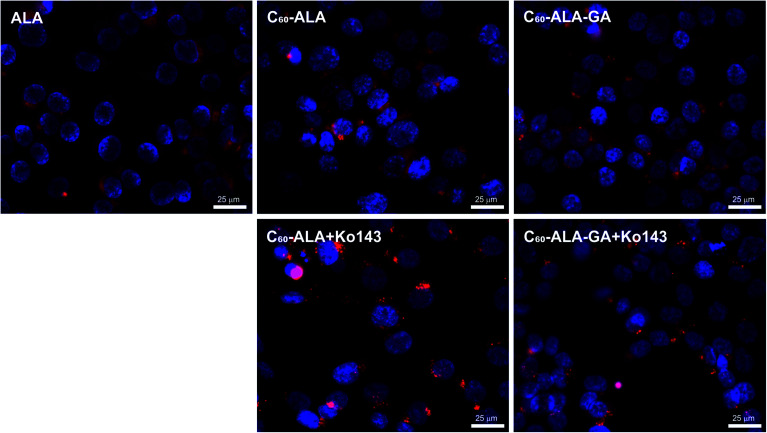
Fluorescence microscopy images of MCF-7 cell line incubated with ALA (1 mg mL^−1^), tested compounds (1 mg mL^−1^), and preincubated with Ko143 (5 μM) after subsequent addition of the tested compounds (1 mg mL^−1^). Scale bar: 25 μm.

In the second PpIX detection approach, the cancer cells were treated with the same concentration of fullerene nanomaterials as in the HPLC analysis; then, after 3 or 24 h, the fluorescence signal of PpIX was measured at 638 nm. In these experiments, we selected three different cancer cell lines. Additionally, Ko143, a specific inhibitor of the ABCG2 (BCRP) multidrug transporter protein, was used to prevent cellular externalization of PpIX. Ko143 has been used in photodynamic therapy to increase the PpIX concentration in cells.^43,44^ After the inhibition of ABCG2, PpIX is not efficiently extracted from the cytosol, and its therapeutic effect is enhanced by the high levels of photosensitizer remaining in the cell. As shown in Fig. 6, the two tested fullerenes were more efficient in increasing the PpIX levels than 5-ALA alone, and C_60_-ALA was slightly more effective in this context. The strongest effect was observed for the A549 cell line, for which the concentration of PpIX increased by more than three times (in comparison with that of ALA alone). For the HCT 116 and MCF-7 cell lines, the photosensitizer levels increased by more than two times. Among the two time points tested, 3 h time was identified as the optimal time because no increase in the PpIX concentration was observed after 24 h. These results highlight the good potential of the tested fullerenes as prodrug carriers for photodynamic therapy. However, we went one step further and considered their combination with an additional component, Ko143. Compared to the untreated cells, a significant increase in PpIX accumulation was observed in MCF-7 and HCT 116 cells treated with the Ko143 inhibitor in combination with 5-ALA and fullerenes after 3 h of incubation. In the case of the A549 cell line, the application of the fullerenes alone resulted in a PpIX level comparable to that reached with the addition Ko143 in the other cell lines. In contrast, no significant increase in PpIX concentration was observed after treatment with Ko143 combined with fullerenes and 5-ALA for the A549 cell line. To confirm these results, we obtained fluorescence microscopy images of PpIX in the cells (Fig. 7). The analysis reveals that cells treated with 5-ALA alone showed the weakest fluorescence intensity. The cells treated with fullerenes showed a stronger signal compared to that of 5-ALA, but weaker than that observed for cells treated with the fullerenes + Ko143 combination, whose fluorescence intensity was the highest. The PpIX accumulation results obtained with the two techniques are consistent with each other.

**Fig. 8:**
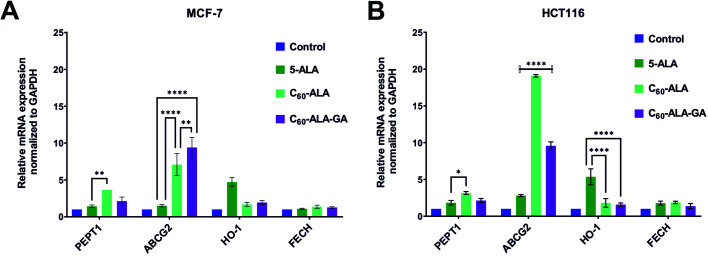
Expression of genes associated with the efficiency of PpIX accumulation 24 h after application of studied compounds to MCF-7 (A) and HCT 116 (B) cell lines. Data were analyzed using one-way ANOVA with Tukey *post hoc* test. *p < 0.05; **p < 0.01; ****p < 0.0001.

In order to assess the combination therapy with Ko143 in higher detail, we performed qRT-PCR experiments. We considered four representative genes, involved in the ALA uptake (*PEPT1*), the incorporation of Fe^2+^ into PpIX (*FECH*), the heme degradation (*HO-1*), and the efflux of PpIX from the cytosol (*ABCG2*). The aim was to determine the initial, middle, and final stages of the heme biosynthetic pathway. Fig. 8 shows dramatically increased levels of *ABCG2* gene after incubation with both fullerenes for both cell lines. The *ABCG2* gene belongs to ATP-binding cassette (ABC) transporters family, responsible for the excretion of excess PpIX from the cell. An increase in ABCG2 expression is a well-known phenomenon that reduces the efficacy of PDT.^13^ Therefore, ABCG2 inhibitors are sought to block PpIX elimination from the cell, increasing its concentration and thus the efficacy of PDT.^45^ The most evident effect, *i.e.*, a 20-fold higher expression of the *ABCG2* gene compared to the control, was observed for the HCT 116 cell line that had been incubated with C_60_-ALA. Intriguingly, the second cell line tested in this experiment (MCF-7) gave a much weaker response, with a 7-fold increase in PpIX levels. In the case of the C_60_-ALA-GA fullerene, the expression showed a 10-fold increase in both investigated cell lines.

These results reveal the specific overexpression of the *ABCG2* gene after addition of 5-ALA or its derivatives, such as the tested fullerenes.^13,46^ We also observed a 5-fold overexpression of the *HO-1* gene after incubation with 5-ALA, which was not detected after incubation with the tested fullerenes. An increased level of *HO-1* gene after incubation with 5-ALA is a common cause of reduced efficacy of PDT; therefore, these results demonstrate that the tested fullerenes had inhibitory properties against HO-1.^47^ The analysis of the *PEPT1* levels revealed a weak overexpression of this gene after incubation with C_60_-ALA in both the tested cell lines. This further confirms the promising potential of this compound as drug carrier. No changes were observed in the *FECH* expression.

**Fig. 9:**
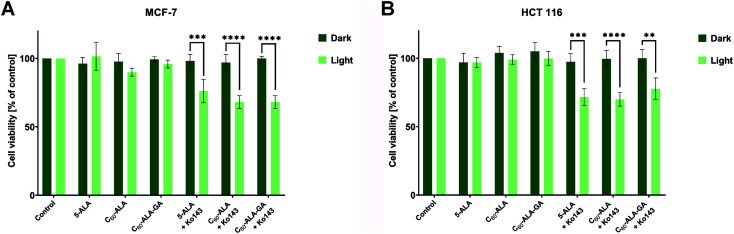
Phototoxicity on MCF-7 (A) and HCT 116 (B) cell lines after 3 h incubation with the tested compounds and the Ko143 inhibitor. The cells were irradiated with red light at 12 J cm^−2^, and the cell viability was determined 24 h after exposure to light. Data were analyzed using one-way ANOVA with Tukey *post hoc* test. **p < 0.01 ***p < 0.001; ****p < 0.0001.

The final step of this study was to investigate the photodynamic effect of the irradiated fullerenes. For this purpose, we incubated the cancer cell lines with C_60_-ALA and C_60_-ALA-GA; after 3 h, the samples were irradiated with red light-excited PpIX. We did not perform PDT studies using blue LED light because of its poor penetration into tumor tissues. Fig. 9 shows the results obtained for the two cancer cell lines considered in this study. The increased levels of the *ABCG2* gene prompted us to evaluate the combination therapy with the ABC transporter inhibitor Ko143. Previous studies by Berg and co-workers described a combination therapy involving 5-ALA and the Ko143 inhibitor, but they used blue light irradiation (405 nm), which has only limited applications on solid tumors in rodent models.^48^ As expected based on the previous experiments (Fig. 6 and 8), no photocytotoxicity was observed for 5-aminolevulinic acid or fullerenes alone when irradiated at 634 nm. However, after adding the ABCG2 inhibitor, the cell survival fraction decreased to approximately 70% in the HCT 116 and MCF-7 cell lines. When analyzing the PDT results, it should be noted that C_60_-ALA and C_60_-ALA-GA contain nine 5-ALA fragments in their structure, which could be further hydrolyzed and metabolized to the appropriate PpIX photosensitizer. After converting concentrations from mg mL^−1^ to mmol L^−1^ (Table S5, ESI†), a similar phototoxicity was observed for all fractions, but the molar concentrations 70% of C_60_-ALA and 24% of C_60_-ALA-GA were equal to a basal concentration of 5-ALA and Ko143 (100%) in the present combination therapy. While these results are encouraging, they suggest that the complex problem of selecting appropriate parameters for cancer PDT requires further in-depth molecular analysis.

## Conclusions

In this study, we successfully fabricated two water-soluble [60]fullerene nanomaterials, namely, C_60_-ALA and its d-glucuronic acid derivative C_60_-ALA-GA. The physicochemical measurements confirmed their structures and stability, showing that nine subunits of 5-ALA were attached to the buckyball core *via* C–N bonds. These carbon nanomaterials delivered a highly effective form of protoporphyrin IX on three cancer cell lines, MCF-7, HTC 116, and A549. The qRT-PCR analyses revealed a significantly higher expression of the *ABCG2* gene (compared to the control) in the HCT 116 cell line that had been incubated with both fullerenes, along with a slight increase in the *PEPT1* gene expression. Furthermore, fullerene-based photodynamic therapy at 634 nm reduced the cell viability to 70% after treatment with the Ko143 inhibitor. The present PDT results highlight the need for further mechanistic studies; additional tests should also assess the effects of different ABCG2 inhibitors or iron chelators.

## Author contributions

Design, conceptualization, and methodology: MS, RG, AMW, RM; chemical synthesis and characterization: MS, MD, MSa, MZ, MSz, ET; cellular experiments: RG, KM; writing and editing: MS, RG, AMW, MD, MSz, RM.

## Conflicts of interest

There are no conflicts of interest to declare.

## Supplementary Material

RA-012-D1RA08499B-s001
