# Supplementary material for: Synthesis and applications of [60]fullerene nanoconjugate with 5-aminolevulinic acid and its glycoconjugate as drug delivery vehicles
Source: RSC Adv. 2022 Feb 22;12(11):6377–88. doi: 10.1039/d1ra08499b (PMC8981668; doi:10.1039/d1ra08499b)
Supplement: RA-012-D1RA08499B-s001 [file RA-012-D1RA08499B-s001.pdf]

Electronic Supplementary Material (ESI) for RSC Advances.
This journal is © The Royal Society of Chemistry 2022

SYNTHESIS AND APPLICATIONS OF [60]FULLERENE NANOCONJUGATE WITH 5-AMINOLEVULINIC ACID AND ITS GLYCOCONJUGATE AS DRUG DELIVERY VEHICLES

Maciej Serda,[a] Robert Gawecki,[b,c] Mateusz Dulski,[d] Mieczysław Sajewicz,[a] Ewa Talik,[c] Magdalena Szubka,[c] Maciej Zubko,[d,e] Katarzyna Malarz,[b,c] Anna Mrozek-Wilczkiewicz,[b,c] Robert Musioł[a]

[a]*Institute of Chemistry, University of Silesia in Katowice, Szkolna 9, 40-006 Katowice, Poland*

[b]*Silesian Center for Education and Interdisciplinary Research, 75 Pulku Piechoty 1a, 41-500 Chorzow, Poland*

[c]*A. Chełkowski Institute of Physics, University of Silesia, 75 Pulku Piechoty 1, 41-500 Chorzow, Poland*

[d]*Institute of Materials Science, University of Silesia in Katowice, 75 Pulku Piechoty, 1A, 41-500, Chorzow, Poland*

[e]*Department of Physics, Faculty of Science, University of Hradec Králové, Rokitanského 62, 500 03 Hradec Králové, Czech Republic*

Correspondence: Dr. Maciej Serda, Institute of Chemistry, University of Silesia in Katowice, Szkolna 9, 40-006 Katowice, Poland. Tel: +48323591545; Fax: +48322599978, email: maciej.serda@us.edu.pl

ELEMENTAL ANALYSIS OF C_{60}-ALA
REACTION SCHEME FOR SYNTHESIS OF C_{60}-ALA and C_{60}-ALA-GA
^{13}C-NMR of C_{60}-ALA
ESI-MS SPECTRUM OF C_{60}-ALA
UV-VIS SPECTRUM OF C_{60}-ALA-GA
DLS AND ZETA POTENTIALS OF C_{60}-ALA and C_{60}-ALA-GA
HPLC MEASUREMENTS OF PpIX IN MCF-7 CANCER CELL LINE
FLUORESCENCE SPECTRUM OF C_{60}-ALA
XPS STUDIES OF C_{60}-ALA-GA
SEQUENCES OF PRIMERS USED FOR RT-PCR

Element	Concentration
C	54.83
O	36.1
N	5.45
H	3.62

Table S1

Elemental analysis of fullerene C_{60}-ALA.

$C_{60}(NHCH_2COCH_2CH_2COOH)_X(OH)_Y$ EMPIRICAL FORMULA

N/C RATIO: $\frac{14X}{720+60X} = \frac{5.45}{54.83}$ X=8.90 ≈ 9 UNITS OF 5-ALA ATTACHED to C_{60}

EMPIRICAL FORMULA: $C_{105}H_{96}N_9O_{51}$ (M_w= 2298 Da)

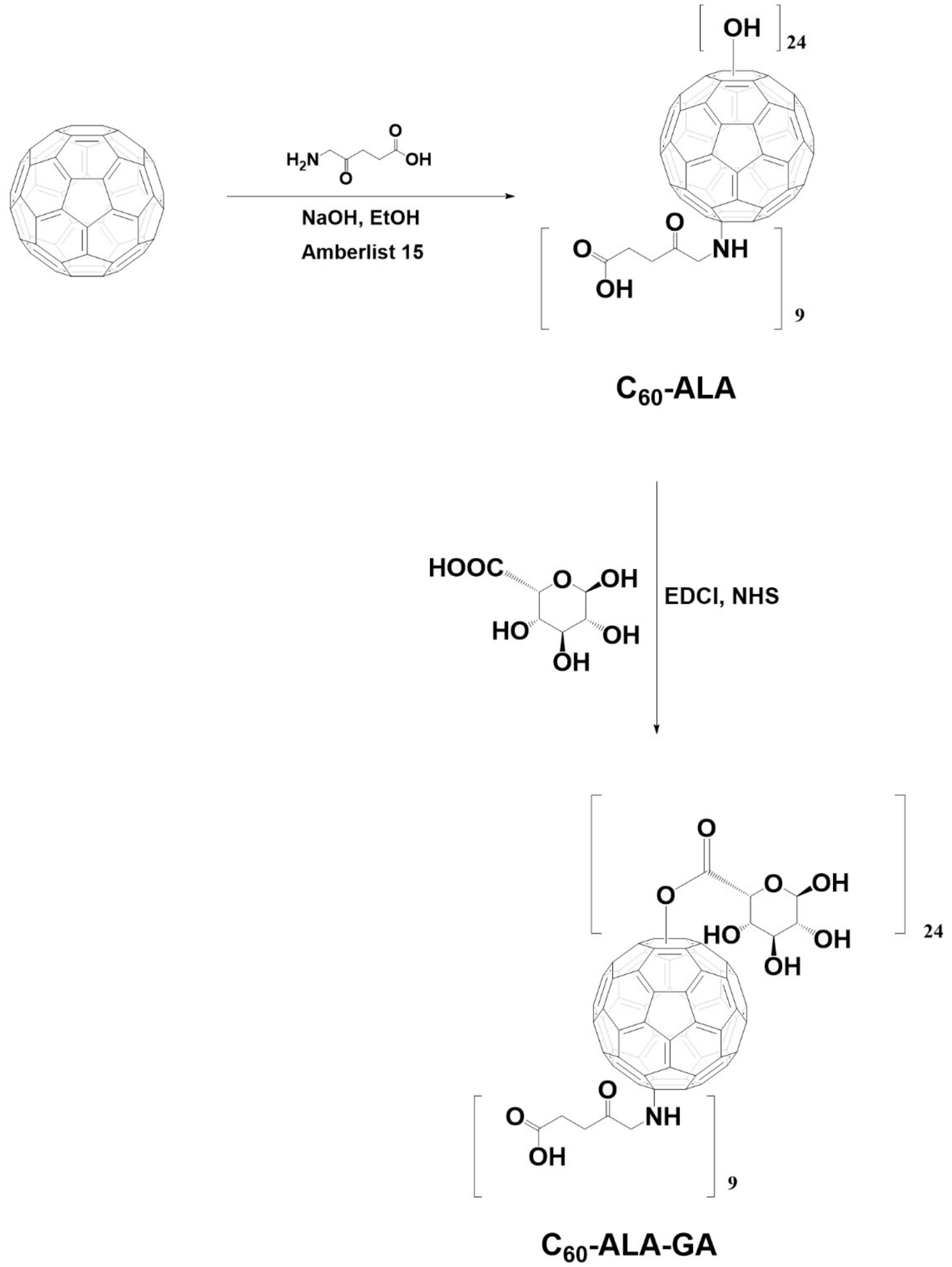

Figure S1

Synthetic plan for obtaining [60]fullerene nanomaterials C_{60}-ALA and C_{60}-ALA-GA

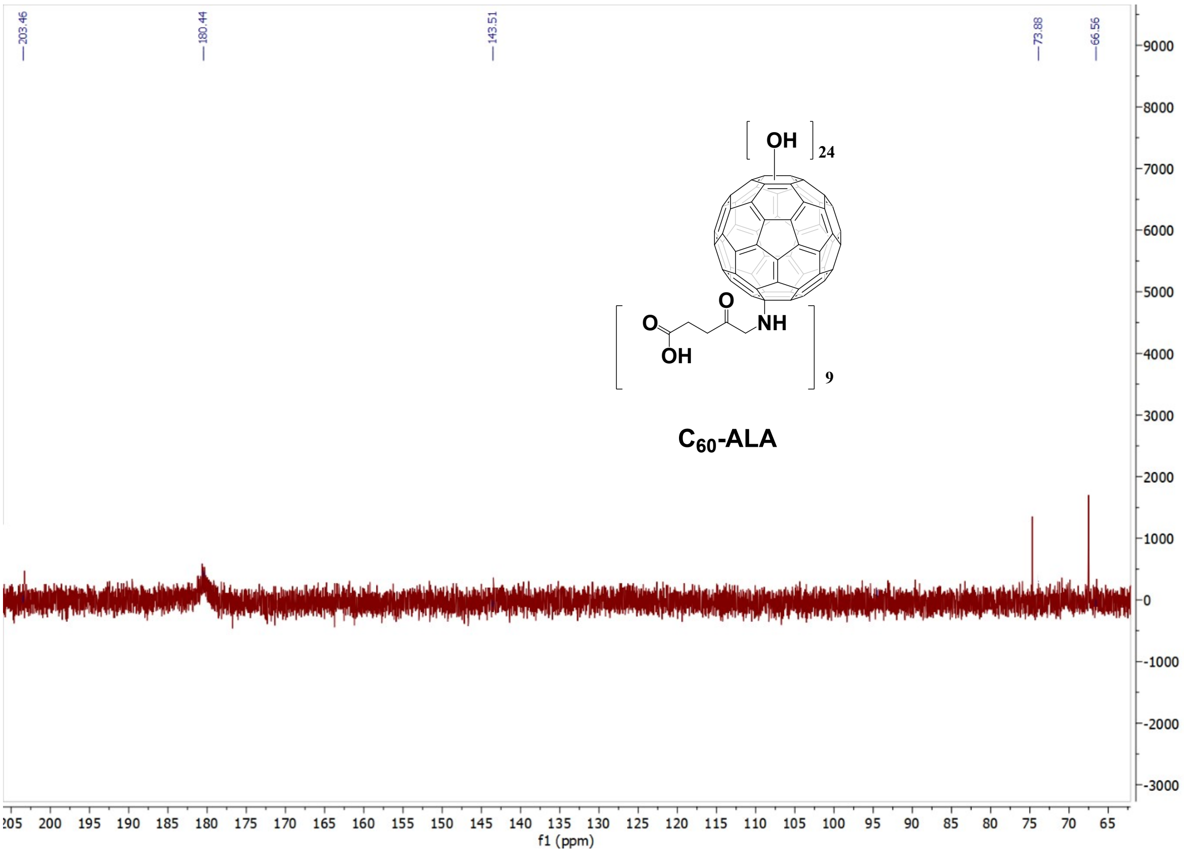

Figure S2

Fragment of ^{13}C-NMR spectrum of fullerene nanomaterial C_{60}-ALA

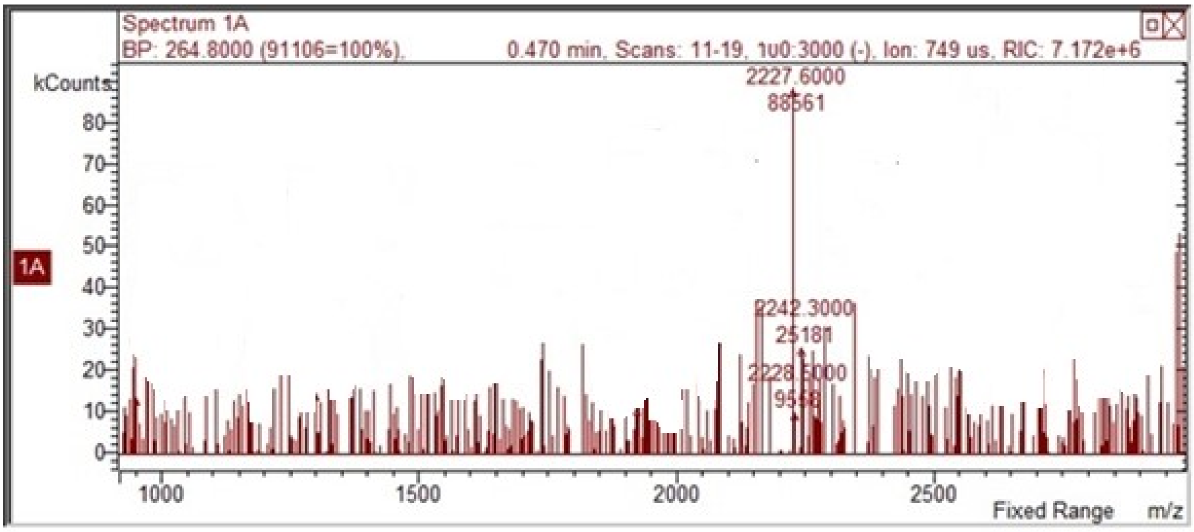

Figure S3

ESI-MS spectrum of fullerene nanomaterial C_{60}-ALA in a positive mode (100 mV).

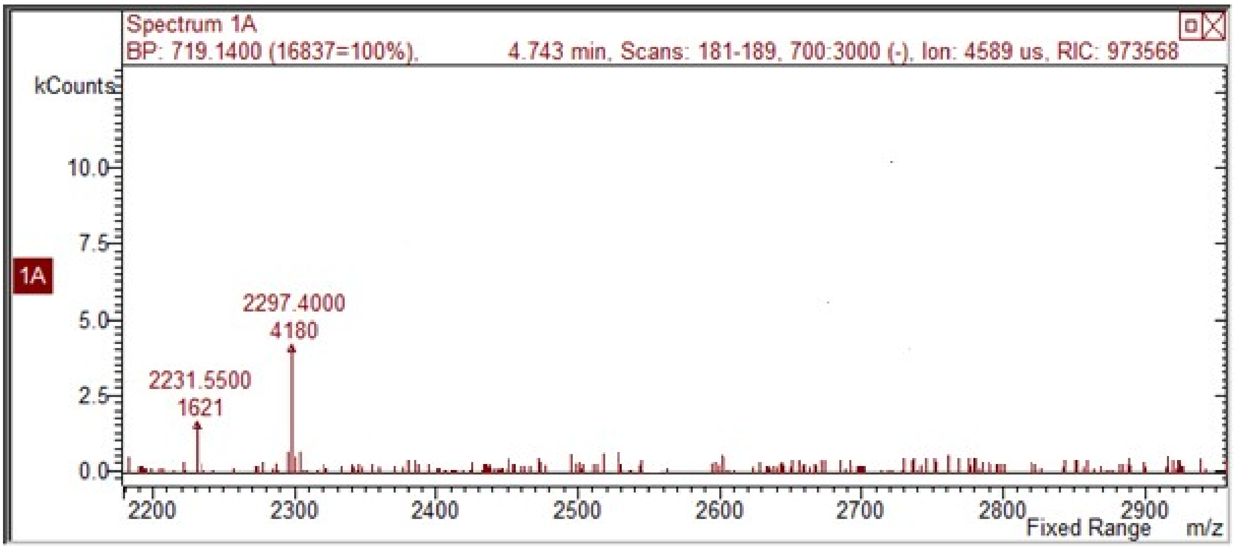

Figure S4

ESI-MS spectrum of fullerene nanomaterial C_{60}-ALA in a negative mode (25 mV).

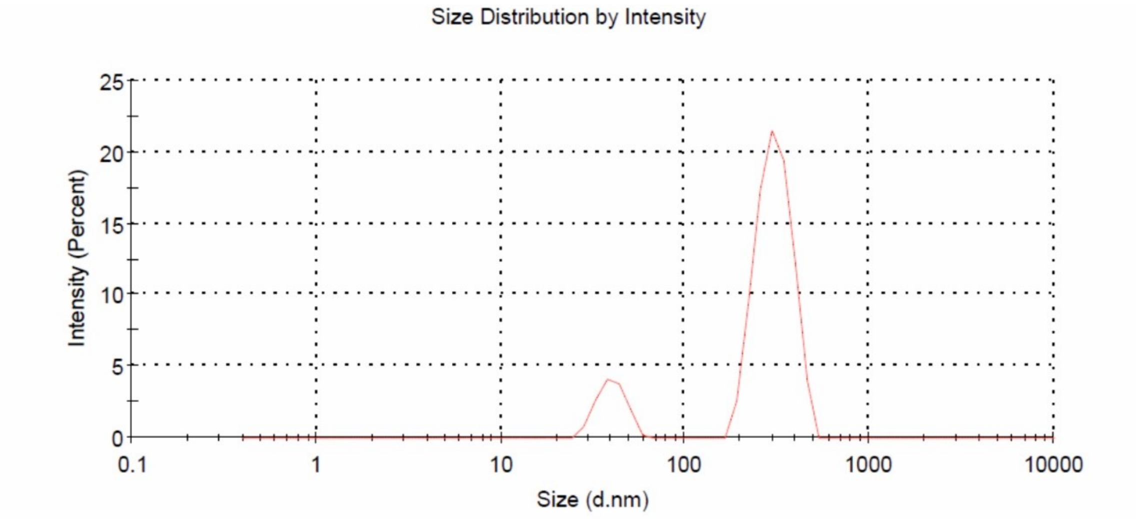

	Size (d.n...	% Intensity:	St Dev (d.n...
**Peak 1:**	308.0	86.4	65.39
**Peak 2:**	40.12	13.6	6.810

Figure S5

Size measurement of fullerene nanomaterial C_{60}-ALA using dynamic light scattering technique.

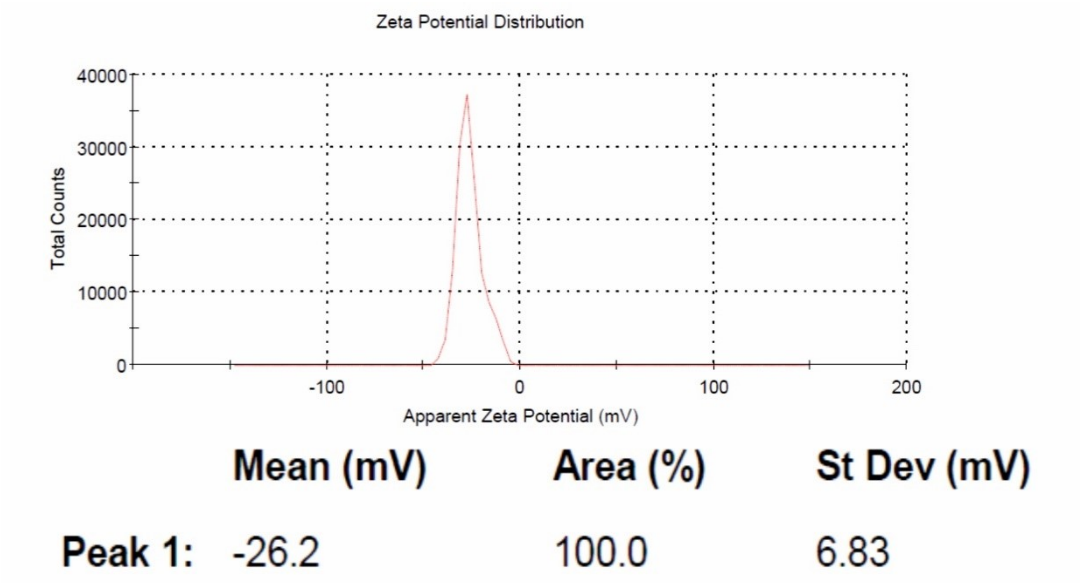

Figure S6

Zeta potential of fullerene nanomaterial C_{60}-ALA.

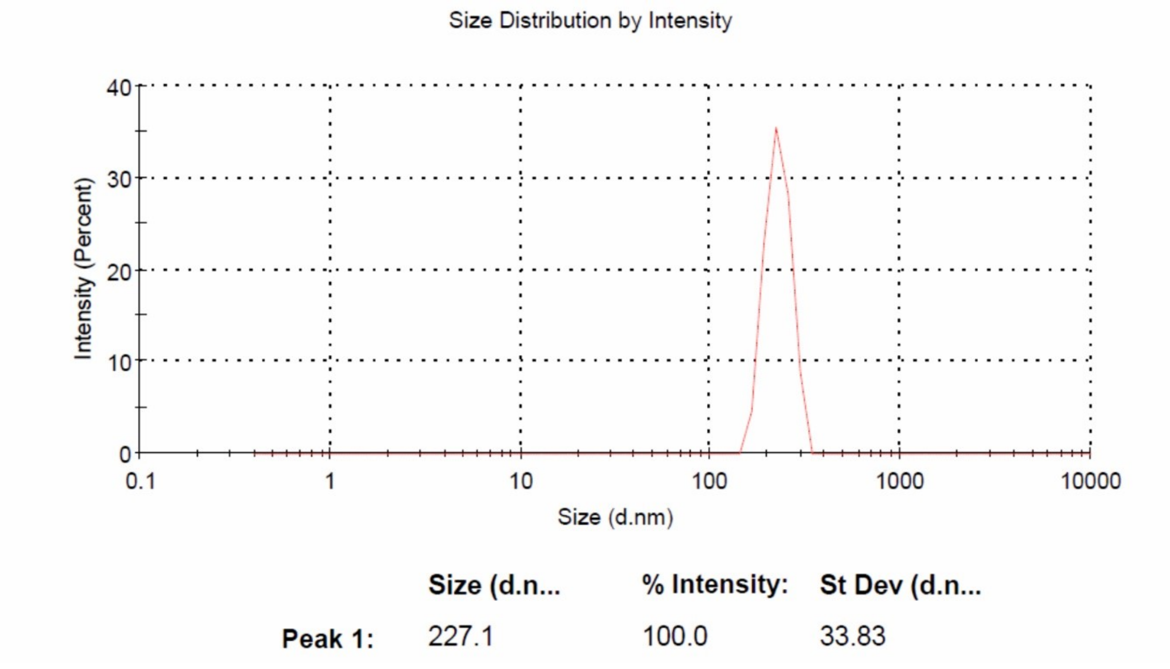

Figure S7

Size measurement of fullerene nanomaterial C_{60}-ALA-GA using the dynamic light scattering technique.

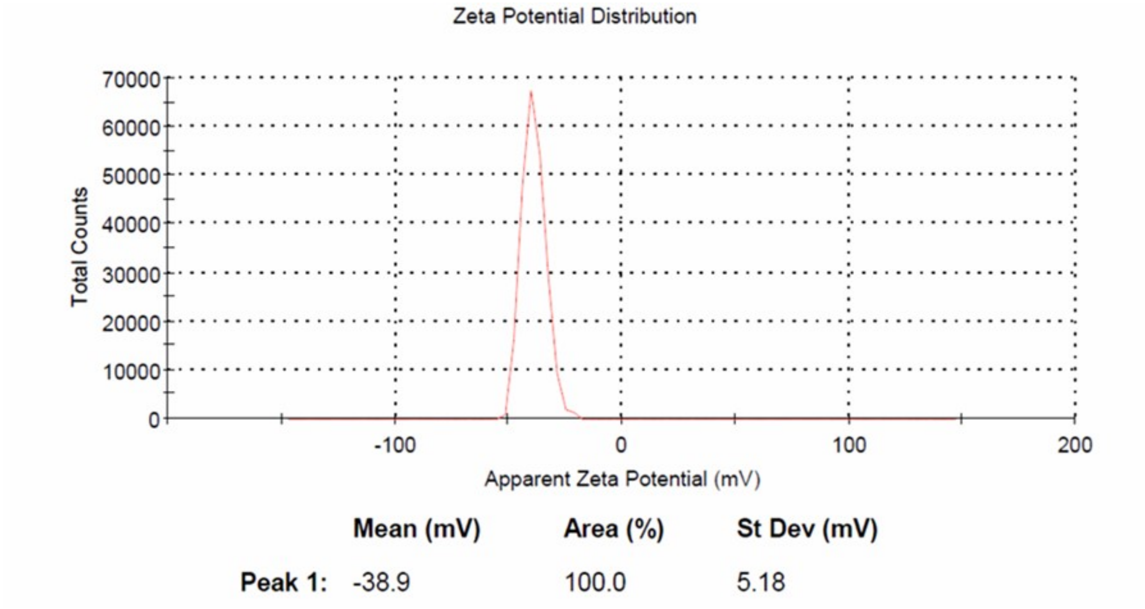

Figure S8

Zeta potential of fullerene nanomaterial C_{60}-ALA-GA.

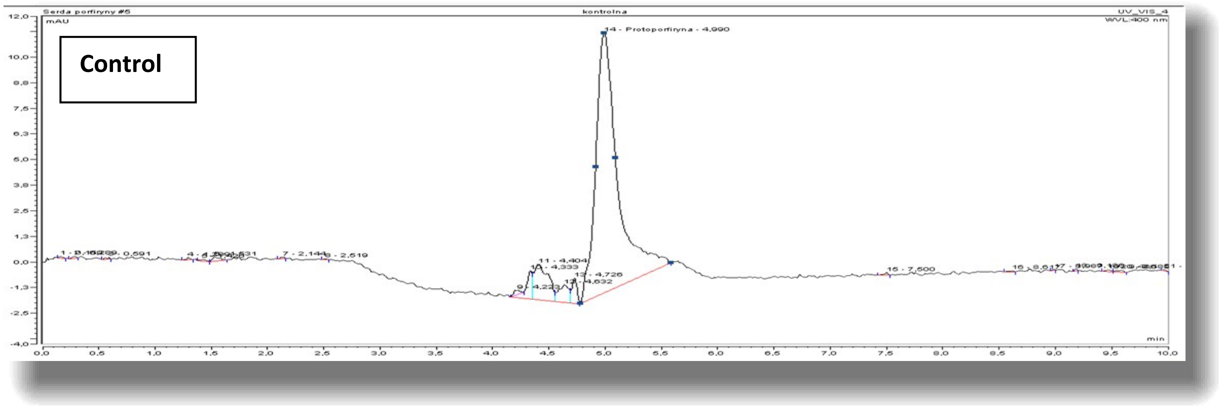
Control
14 - Protoporfiryna - 4,990
mAU
min

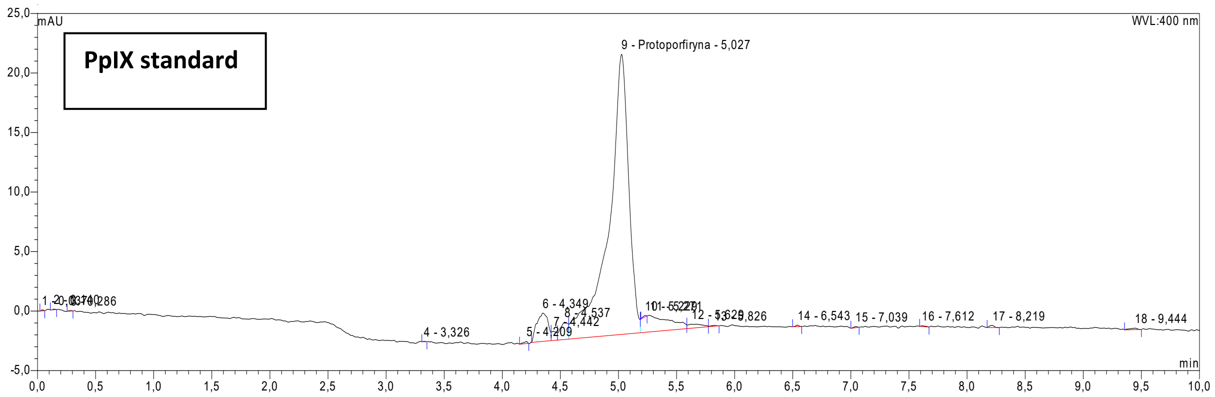
PpIX standard
9 - Protoporfiryna - 5,027
mAU
WVL:400 nm
min

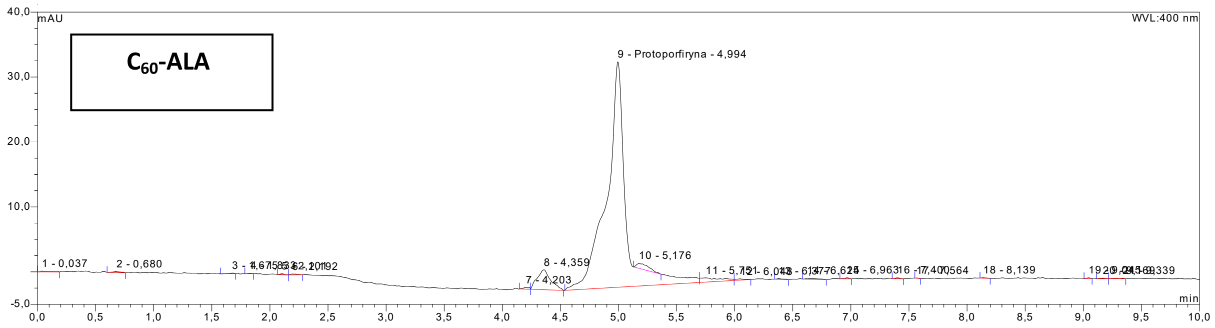
C60-ALA
9 - Protoporfiryna - 4,994
mAU
WVL:400 nm
min

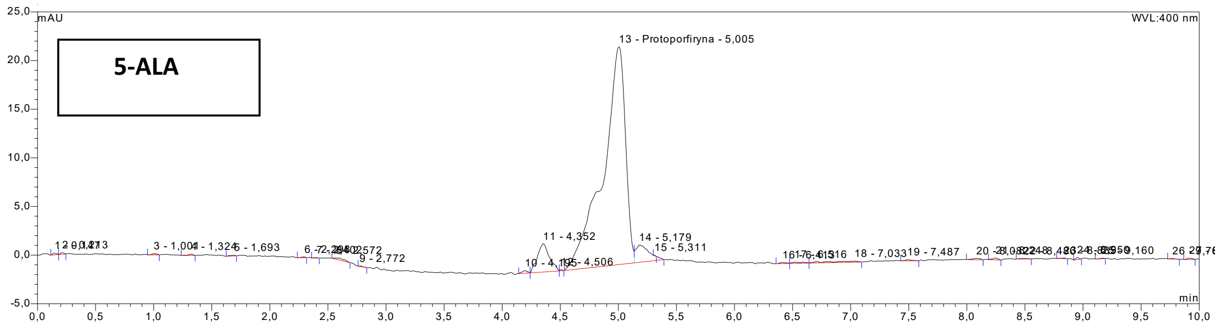
5-ALA
13 - Protoporfiryna - 5,005
mAU
WVL:400 nm
min

Figure S9

The HPLC chromatograms measured for dedicated bio-extracts from MCF-7 cancer cell line and standard (PpIX, Sigma Aldrich). The cells before extraction were untreated (control) or treated with 5-ALA, or fullerene nanomaterial C_{60}-ALA.

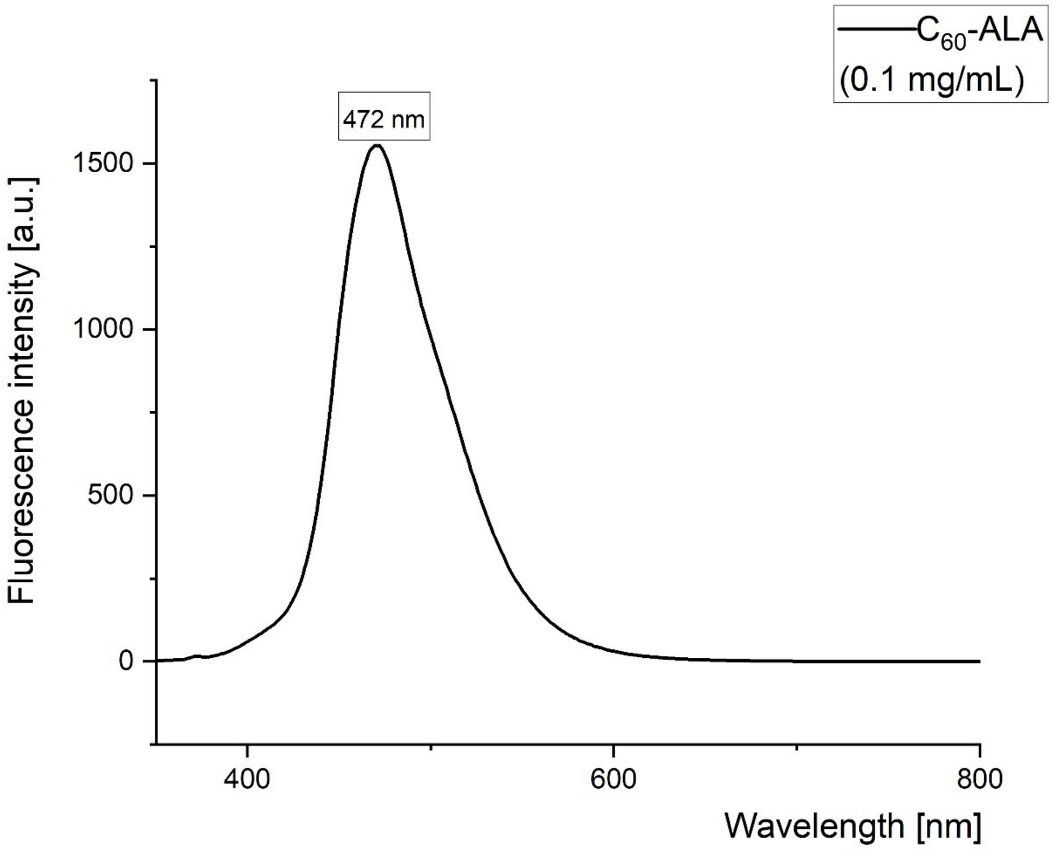

Figure S10

Fluorescent spectrum of C_{60}-ALA in water (c=0.1 mg/mL) excited at 340 nm.

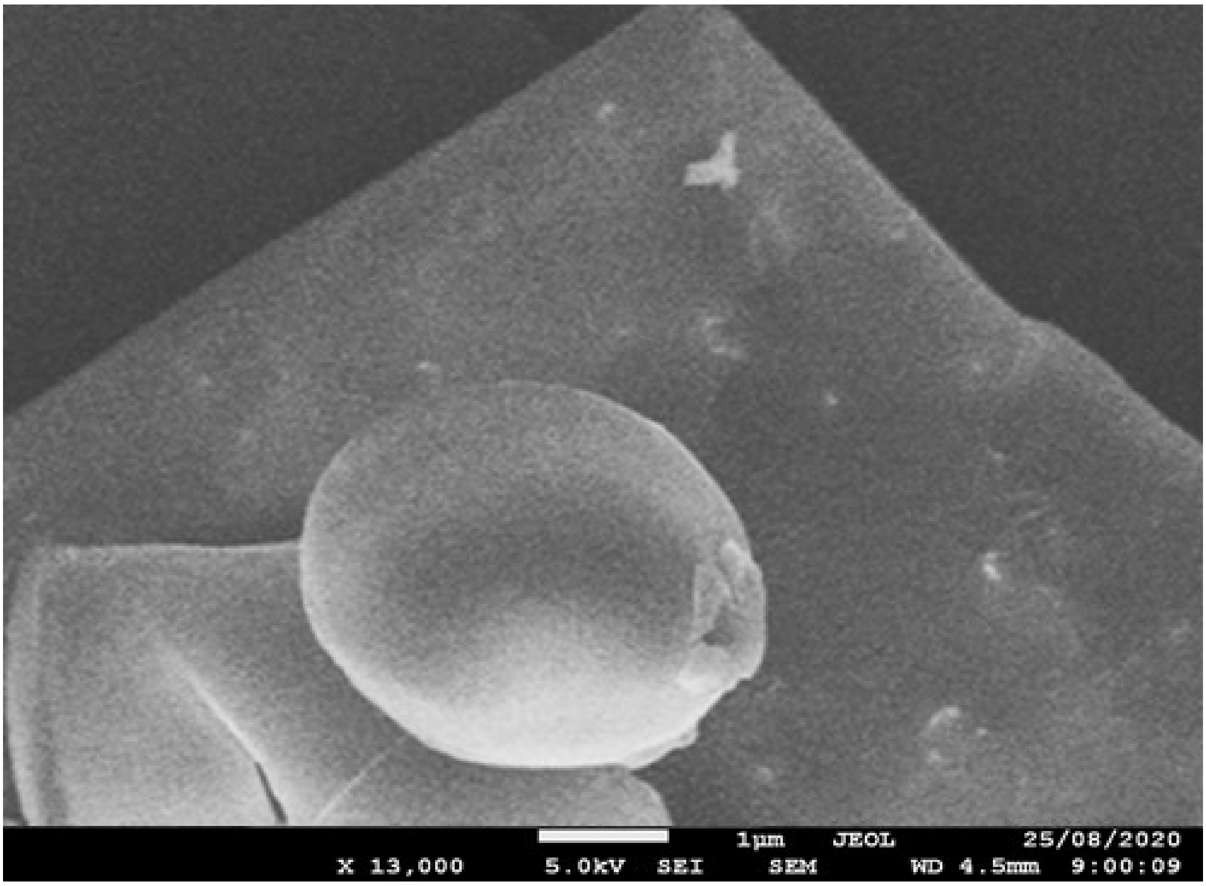

Figure S11

SEM image of fullerene nanomaterial C_{60}-ALA confirming its hollow structure.

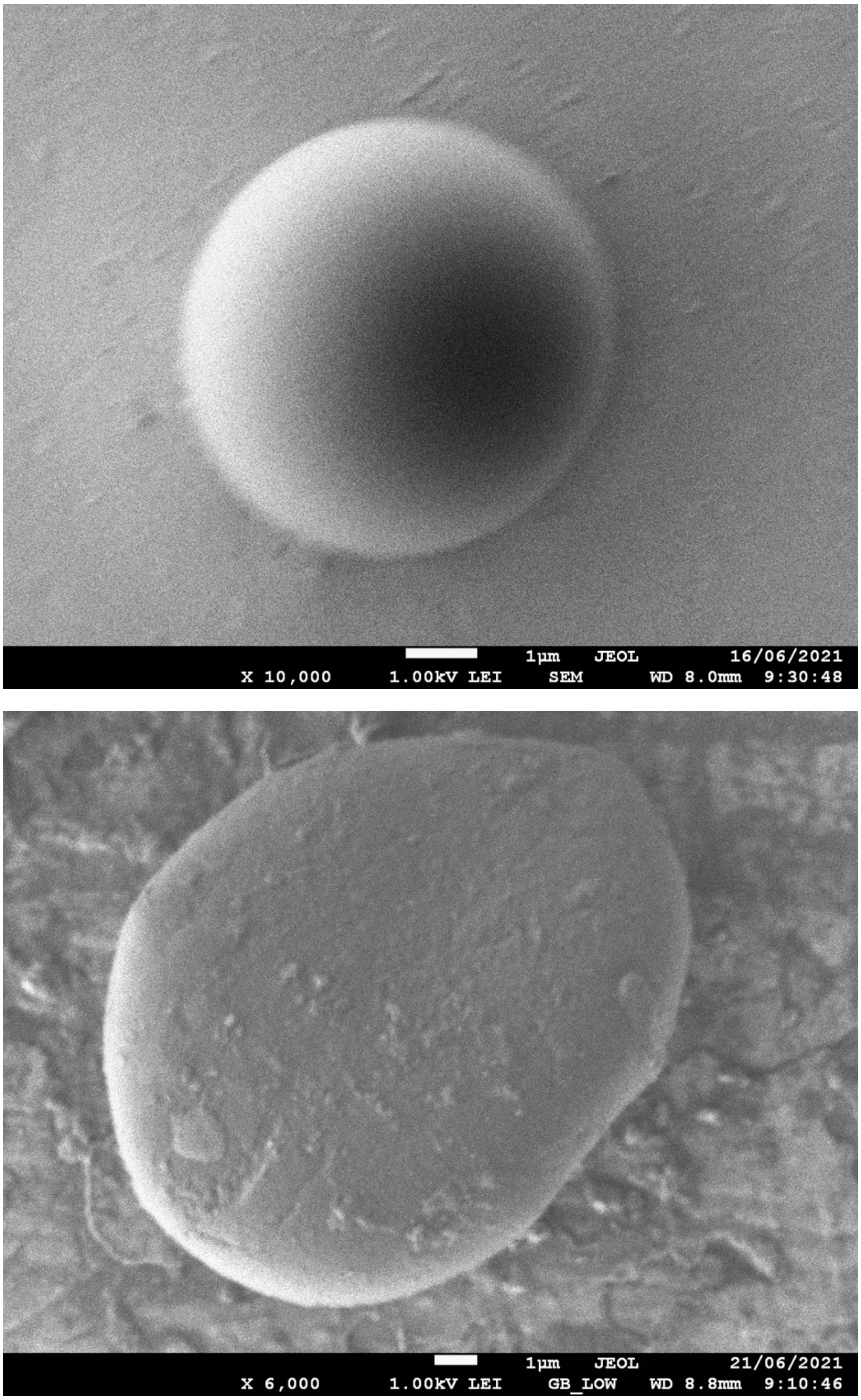

Figure S12

SEM images of fullerene nanomaterial C_{60}-ALA-GA.

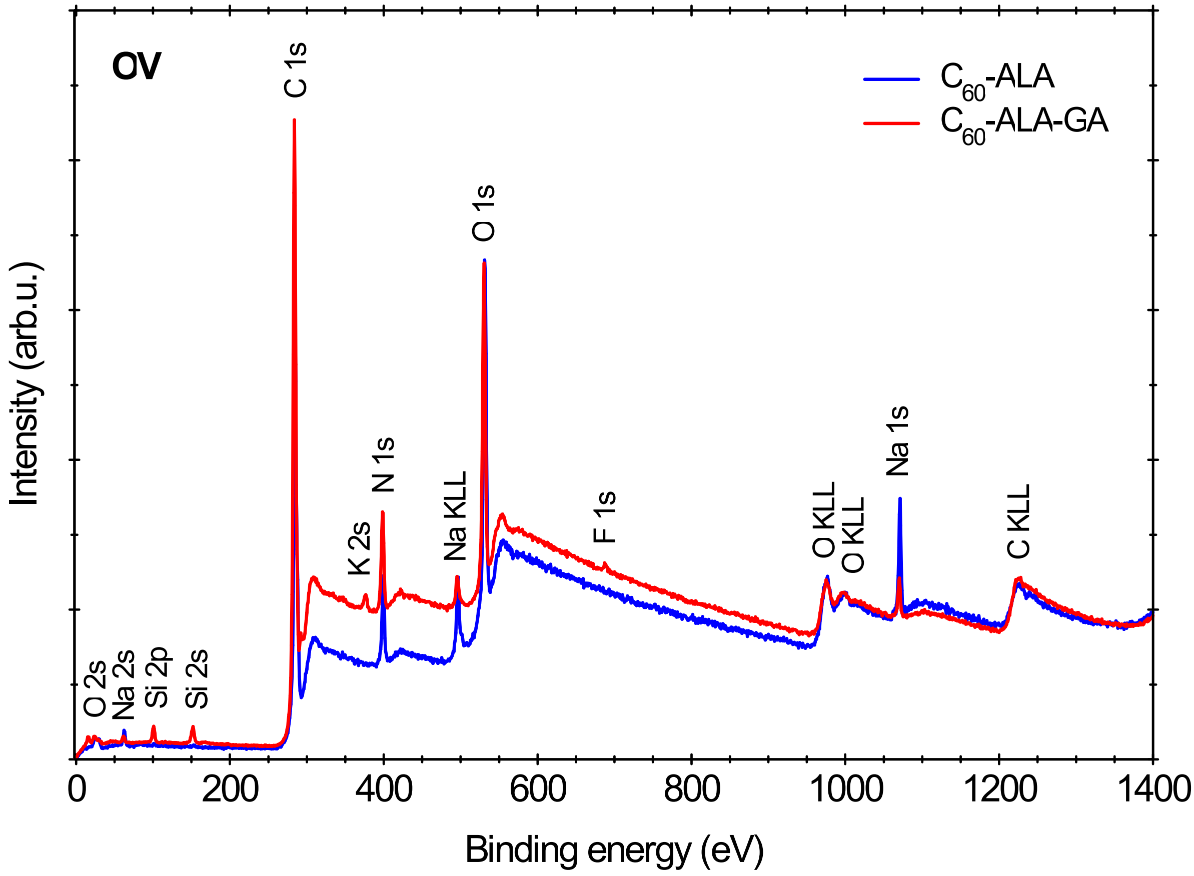

Figure S13

The XPS spectra of two fullerene nanomaterials in the wide energy range 0–1400 eV.

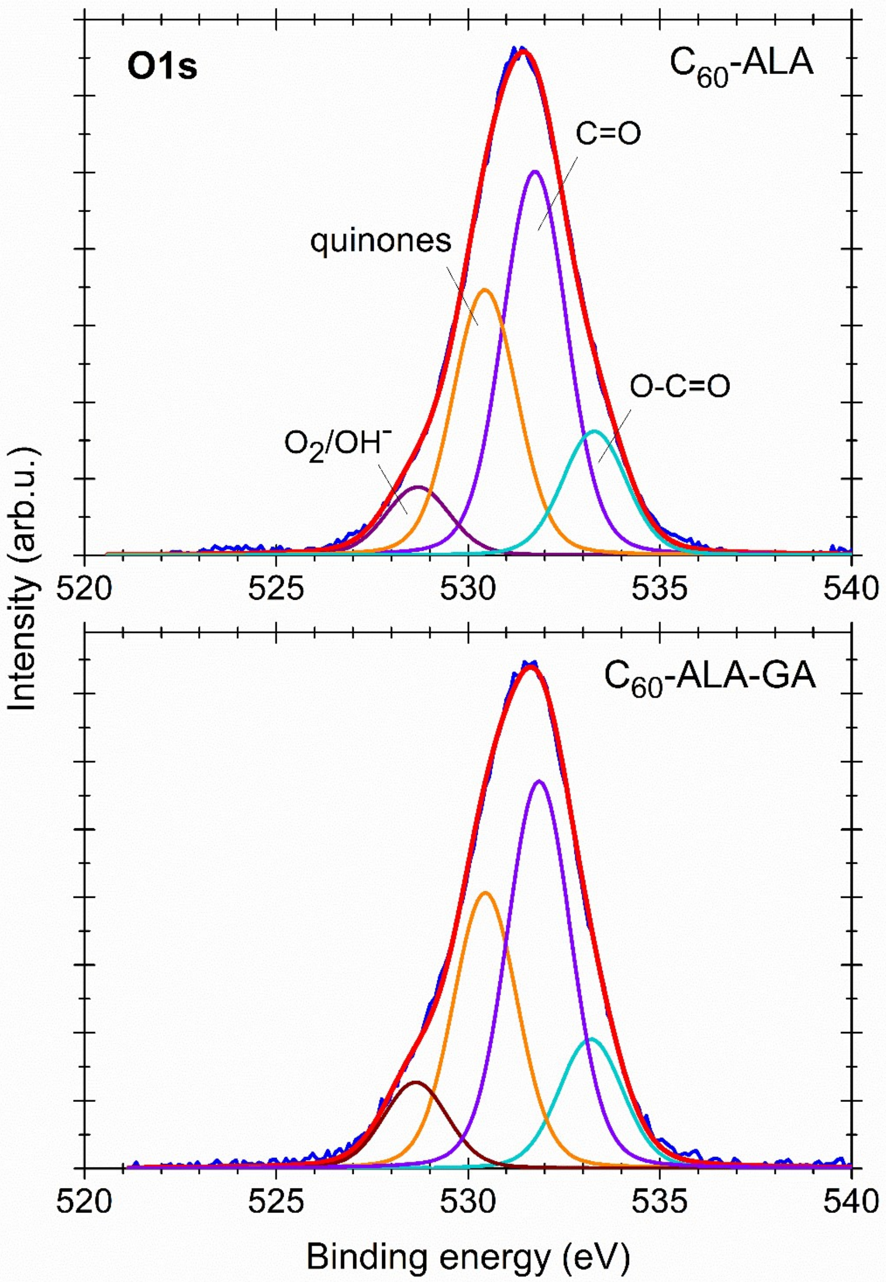

Figure S14

A high-resolution photoemission spectra of oxygen (O1s) measured for selected fullerene nanomaterials C_{60}-ALA and C_{60}-ALA-GA.

Compound	C (mg/mL)	MCF 7 (mg/mL)	A549 (mg/mL)	HCT116 (mg/mL)
CONTROL	0	0.37	0.41	0.78
5-ALA	1	0.67	0.47	1
C_{60}-ALA	1	0.71	0.66	1.34
C_{60}-ALA-GA	1	1.52	1.6	3.38

Table S2

Quantitative HPLC of protoporphyrin IX detected in the cellular lysates and measured at 400 nm after the treatment of cells with 5-ALA or fullerene nanomaterials. The MCF7, A549 and HCT116 cancer cell lines were used in this study. The final results were normalized to number of substrate mmols.

	C_{60}-ALA				C_{60}-ALA-GA			
**Element**	**Atomic concentration [%]**	**Chemical states**	**Binding energy [eV]**	**Contributions of lines [%]**	**Atomic concentration [at %]**	**Chemical states**	**Binding energy [eV]**	**Contributions of lines [%]**
C1s	64.3	C-Si	281.53	3.7	69.9	C-Si	281.54	3.7
		C=C	283.23	17.7		C=C	283.21	17.8
		C-H, C-C	284.89	55.4		C-H, C-C	284.90	51.1
		C-O, C-N, -C-OH	286.41	11.4		C-O, C-N, -C-OH	286.36	17.7
		O=C-OH, C=O	288.27	11.8		O=C-OH, C=O	288.28	9.8
O1s	24.0	O_2/OH^-	528.69	8.1	17.7	O_2/OH^-	528.68	10.5
		quinones	530.46	31.5		quinones	530.49	32.1
		C=O	531.89	45.6		C=O	531.93	44.7
		O-C=O	533.26	14.8		O-C=O	533.28	12.7
N1s	7.8	C-N, N-(C=O)-	398.43	31.4	8.1	C-N, N-(C=O)-	398.35	34.4
		pyridinic N	399.79	60.9		pyridinic N	399.76	54.4
Na1s	3.5	-	-	-	1.1	-	-	-
Si2p	0.2	-	-	-	1.7	-	-	-
K2p	-	-	-	-	1.1	-	-	-
F1s	-	-	-	-	0.2	-	-	-
Cl2p	0.1	-	-	-	0.1	-	-	-

S2p	0.1	-	-	-	0.1	-	-	-

Table S3

Chemical composition and atomic concentrations for C_{60}-ALA and C_{60}-ALA-GA, determined from XPS measurements.

Gene name	***Sequence of primer***	
**PEPT1**	F	AGGCAACAACTATGTCCGGG
	R	CACAGCATCGAAGATCGGGA
**ABCG2**	F	GCACAGGAAGTTTACGCACAG
	R	AAGGGGCTAGAAGAAGGGGG
**FECH**	F	GATGAATTGTCCCCCAACAC
	R	GCTTCCGTCCCACTTGATTA
**HO-1**	F	CATCCCCTACACACCAGCCA
	R	ATGTTGGGGAAGGTGAAGAAGG
**GAPDH**	F	GAGTCAACGGATTTGGTCGTA
	R	GCCCCACTTGATTTTGGAG

Table S4

The sequences of the primers used in RT-PCR experiments.

Compound	C [mg/mL]	C [mmol/L]	C_{max} 5-ALA
C_{60}-ALA	1	0.464	4.176
C_{60}-ALA-GA	1	0.157	1.413
5-ALA	1	5.97	5.97

Table S5

The conversion of concentration units for selected fullerene nanomaterials.
